# Photoreception and transcriptomic response to light during early development of a teleost with a life cycle tightly controlled by seasonal changes in photoperiod

**DOI:** 10.1371/journal.pgen.1010529

**Published:** 2022-12-12

**Authors:** Mariann Eilertsen, David W. P. Dolan, Charlotte M. Bolton, Rita Karlsen, Wayne I. L. Davies, Rolf B. Edvardsen, Tomasz Furmanek, Harald Sveier, Herve Migaud, Jon Vidar Helvik

**Affiliations:** 1 Department of Biological Sciences, University of Bergen, Bergen, Norway; 2 Department of Informatics, University of Bergen, Bergen, Norway; 3 Institute of Aquaculture, University of Stirling, Stirling, Scotland, United Kingdom; 4 Umeå Centre for Molecular Medicine, Umeå University, Umeå, Sweden; 5 School of Life Sciences, College of Science, Health and Engineering, La Trobe University, Melbourne, Australia; 6 Institute of Marine Research, Bergen, Norway; 7 Lerøy Seafood Group ASA, Bergen, Norway; Universidad de Valparaiso, CHILE

## Abstract

Light cues vary along the axis of periodicity, intensity and spectrum and perception of light is dependent on the photoreceptive capacity encoded within the genome and the opsins expressed. A global approach was taken to analyze the photoreceptive capacity and the effect of differing light conditions on a developing teleost prior to first feeding. The transcriptomes of embryos and alevins of Atlantic salmon (*Salmo salar*) exposed to different light conditions were analyzed, including a developmental series and a circadian profile. The results showed that genes mediating nonvisual photoreception are present prior to hatching when the retina is poorly differentiated. The clock genes were expressed early, but the circadian profile showed that only two clock genes were significantly cycling before first feeding. Few genes were differentially expressed between day and night within a light condition; however, many genes were significantly different between light conditions, indicating that light environment has an impact on the transcriptome during early development. Comparing the transcriptome data from constant conditions to periodicity of white light or different colors revealed overrepresentation of genes related to photoreception, eye development, muscle contraction, degradation of metabolites and cell cycle among others, and in constant light, several clock genes were upregulated. In constant white light and periodicity of green light, genes associated with DNA replication, chromatin remodeling, cell division and DNA repair were downregulated. The study implies a direct influence of light conditions on the transcriptome profile at early developmental stages, by a complex photoreceptive system where few clock genes are cycling.

## Author summary

Light conditions, including, periodicity, intensity and spectrum, are environmental factors that have driven evolution and shaped the organisms photosensory system and regulation of biological processes from behavior to gene expression. In this study, we have taken advantage of early development in Atlantic salmon, an organism with life history closely linked to changing light environment, that develop to an advance stage prior to first feeding. The data represent a long-term exposure trail of 4 months, which is independent of disturbing effects of feeding, giving the opportunity to gain insight into the direct effects of different light conditions on the transcriptome profile. The transcriptomic data reveal that the photoreceptive capacity and a non-cycling clock system develop early. Overall, many genes representing various pathways were significantly different between light conditions. In our opinion, this holistic dataset and results represent a very important step to gain better overview of how different light conditions impact the transcriptome during development.

## Introduction

Light is one of the most important environmental cues known to modulate the behavior, physiology and gene expression of organisms. In nature, the daily solar cycle provides a predictable rhythm of light and dark periods that allows for the entrainment and regulation of many circadian and circannual biological processes by non-image forming photoreception [1,2]. Knowledge of how an organism perceives, processes and integrates light cues to entrain and regulate biological rhythms and physiological events is essential for understanding how the genotype- phenotype axis is influenced by different light environments. With this information, it may be possible to determine or predict which light conditions are essential for development, growth and reproduction of a particular species.

The presence of photopigments in mammals was for many years associated with retinal tissue, such as the rods, cones and the intrinsically photosensitive retinal ganglion cells [3], but lately the non-image forming photoreception has been shown to be far more complex [4–6]. In nonmammalian vertebrates, the perception of light is predominantly active in the retina, pineal organ and in several regions of the deep brain [3,7,8] although the discovery of light-sensitive molecules in other tissues (e.g. skin, heart, intestine) suggests that photoreception is likely to be far more widespread [9]. In the past decade, the complexity of non-classical photoreceptive systems has expanded, e.g. with the discovery of 32 nonvisual opsins in zebrafish (*Danio rerio*) [9]. Interestingly, the array of nonvisual opsins is even more complex in Atlantic salmon (*Salmo salar*) due to the salmonid-specific fourth whole genome duplication, Ss4R [10–12].

In nature, salmon eggs hatch and develop in the river gravel during the winter months before the alevins emerge and feed in the river during the spring and these life history transitions are controlled by the photoperiod. Alevins kept under different light/dark regimes have well synchronized emergence from the river gravel in the dark period [13]. The anadromous life cycle of salmon is tightly controlled by seasonal changes in photoperiod [14–16] and salmon is therefore an excellent model for understanding photoreception and the impact of light on development. Studies revealing the function of nonvisual opsins in fish are however sparse, but the expression of nonvisual opsins has been described in some fish species during early development [17–19], and in juvenile and adults [8,20–23]. In zebrafish larvae, it has been suggested that melanopsin-expressing cells in the preoptic region regulate a light-seeking behavior triggered by loss of illumination [24] and in zebrafish embryos prior to hatching, photoreceptors in the hindbrain were shown to be responsible for a “photomotor response” after exposure to a bright light stimulus [25]. In Atlantic halibut (*Hippoglossus hippoglossus*), a transient cluster of dual photoreceptors in the hindbrain was indicated to be responsible for the light regulated hatching process [26]. Transcriptome activation can be regulated directly through such light stimulation of photoreceptors or through an endogenous timekeeper, the circadian clock, consisting of transcriptional-translational feedback loops composed of clock genes [27–29].

The effect of light on fish biology has been studied in several species by exposing fish embryos and larvae to different light environments during development [30,31]. For example, zebrafish larvae raised under different light periodicity and wavelengths revealed that the hatching rate is highest under light-dark periodicity (LD) of blue and violet wavelengths, and that constant light leads to a higher proportion of malformation [32]. Zebrafish larvae exhibited enhanced growth in LD with violet and blue wavelengths, which was supported by significantly higher expression of several growth factors [32]. Further, Atlantic cod (*Gadus morhua*) and Atlantic turbot (*Scophthalmus maximus*) larvae exposed to blue and green light showed significantly enhanced growth rates compared to larvae exposed to red light, even though green light appeared to reduce the survival rate for both species [33]. The consensus for marine species seems to be that blue and green wavelengths matching the natural environment have a positive effect on fish welfare when analyzing behavioral responses such as stress, locomotor activity, feed intake and reproduction [32]. The phenotypic responses reported in these studies are a result of the overall expression of many genes that are directly or indirectly regulated by light. However, the underlying mechanisms and pathways remain unknown. Microarray analyzes of gene expression in fish studying the effect of light shifts, have provided some interesting findings. For example, a circadian sampling series in feeding gilthead sea bream larvae (*Sparus aurata*) revealed a diurnal activation of pathways related to phototransduction, intermediary metabolism, development, chromatin remodeling and cell cycle regulation [34]. In zebrafish larvae, a shift from darkness to light revealed an enrichment of genes involved in circadian rhythms, stress response and DNA repair [35]. These studies indicate that the light environment is perceived early and may regulate important pathways for development, such as cell cycle, metabolism and DNA repair.

Many of the light stimulation studies in fish were performed after first feeding in marine larvae [33,34,36] with an aim to improve fish performance and the number of hatchlings given the very low survival rates generally reported in these species. The present study was performed on Atlantic salmon given its many advantages for developmental and circadian research e.g. numerous large demersal eggs, very high survival rates, long developmental window, absence of metamorphosis, hatching of large alevins, strong seasonality, as well as the availability of high quality genomic and transcriptomic data. Taking a global approach, the aim of this study was to reveal the photoreceptive capacity and the effect of light conditions on the developing Atlantic salmon prior to first feeding, when the alevins are not dependent on exogenous feeding. A four-month light exposure experiment was performed with Atlantic salmon embryos from fertilization to the alevin stage, including a developmental and a circadian series to obtain an overview of the photoreceptive capabilities and cycling dynamics. The results showed a complex photoreceptive system and that most nonvisual opsins were expressed prior to hatching and even though many clock genes were expressed, few were cycling. The analyses showed that many genes were significantly different between light conditions, indicating that light environment has an impact on the transcriptome during early development.

## Results

This study represents a long-term development series (up to 113 days) under different light conditions where embryos and alevins have been analyzed from end of the somitogenesis to an advanced developmental stage just prior to first feeding (S1A Fig).

### Transcriptome development, photoreceptive capacity and the circadian clock during development

**Fig 1:**
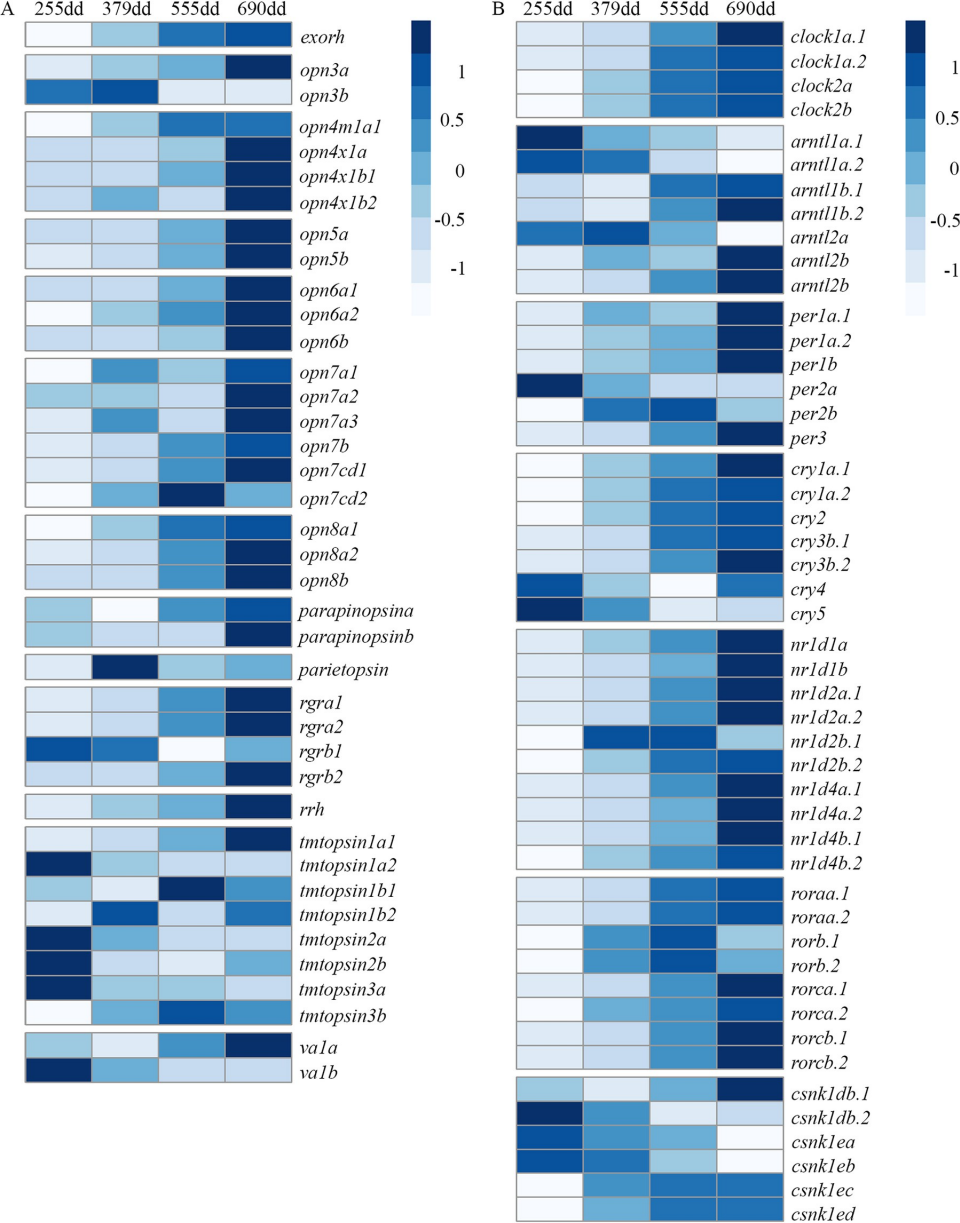
Heatmap of nonvisual opsins and clock genes in Atlantic salmon. The heatmap, scaled by row, shows the average normalized counts for nonvisual opsins (see S3 Table for annotations) and clock genes (see [38] for annotations) in the developmental series of embryos and alevins reared in light-dark cycles of medium intensity (LDM). Genes not expressed or not annotated at Ensembl were not included. The heatmaps show that many nonvisual opsins and clock genes are expressed early in development.

At the first developmental stage studied, 255 dd, the somitogenesis was competed and the eye pigmentation had just occurred. The two next stages (379 and 555 dd) were in the life history transition of hatching, before and after, respectively, while the last stage (690 dd) correspond to the free swimming alevin prior to first feeding [37]. In S2 Fig the transcriptomic dynamics of the developing embryo and alevin are described, showing that many genes were expressed already at 255 dd (S2A Fig), but that many genes were differentially expressed compared to alevins prior to first feeding (690 dd) (S2B Fig), highlighted by gene ontologies related to development of the embryo (e.g. brain development and mitotic cell cycle, RNA processing and transcription) (S2C Fig and S4 Table). Fig 1 and S5 Table reveals the photoreceptive and circadian clock capacity of the developing embryos and alevins. The heatmap shows that some of the nonvisual opsin classes (e.g. *exorh*, *opn3*, *rgr*, *rrh*, *tmtopsin*, *va* opsin) were expressed early and as development progressed, almost all were expressed prior to first feeding (Figs 1A and S3 for individual counts). All clock genes [38] were expressed at 255 dd with increased expression as development progressed (see Figs 1B and S4 for individual counts). A logfold2 change heatmap (S5 Fig) shows that the greatest change in expression was for the clock genes between 255 dd and 379 dd. Of the 42 nonvisual opsins, considered to be functional, there were 14 Ss4R ohnologue pairs and 14 single copy genes (S3 Table). The heatmaps show that expression of ohnologues clearly differed for opsin classes, e.g. vertebrate ancient opsin, *va1b* was highly expressed already at 255 dd while *va1a* is lowly expressed.

### Cycling genes under light-dark transitions

**Fig 2:**
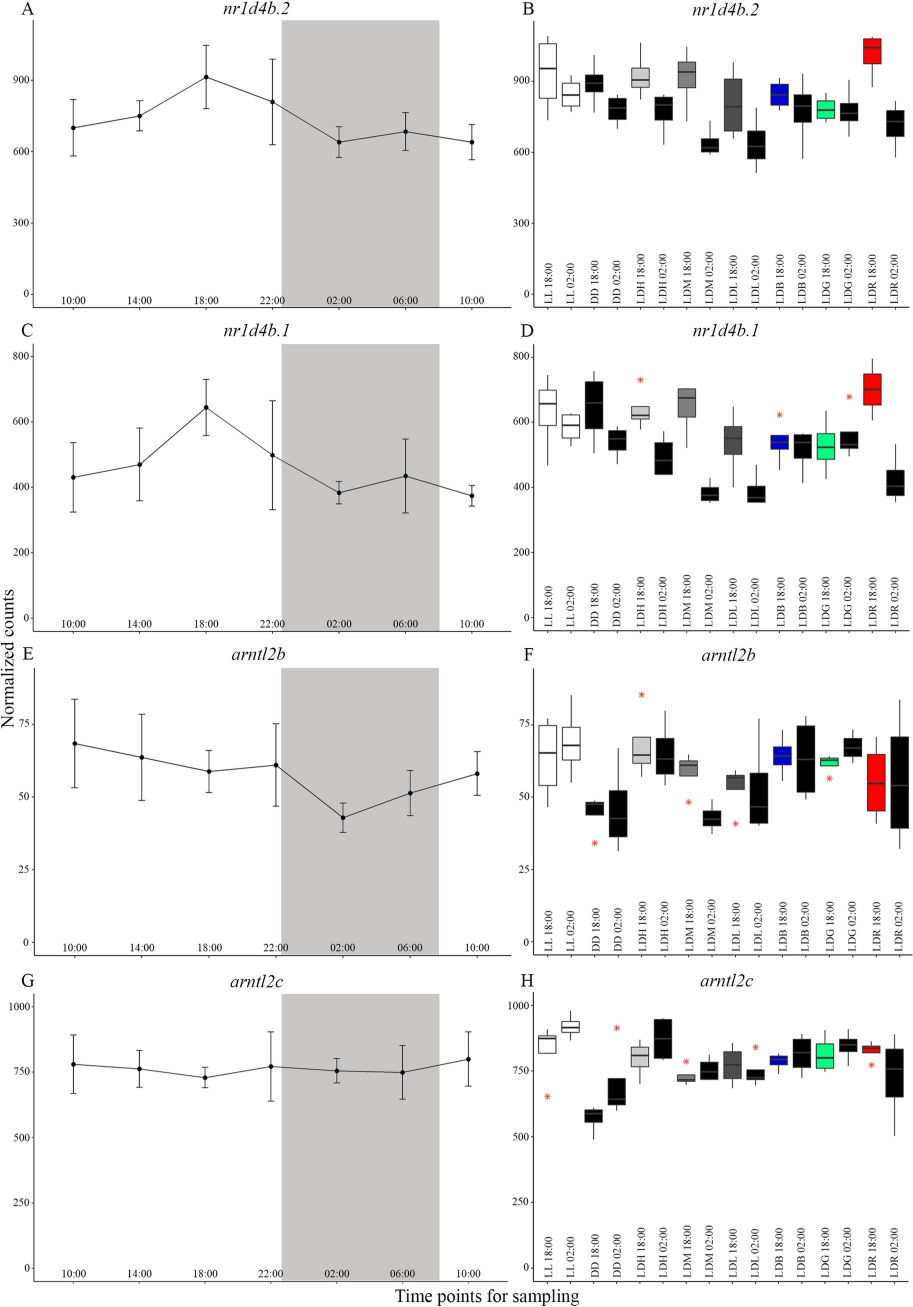
Clock genes and their ohnologues observed as cycling by Kendall’s rank correlation coefficient in JTK_CYCLE. A, C, E, G) The normalized counts for the circadian series under light-dark of medium intensity of white light (LDM) conditions plotted as standard deviation. B, D, F, H) Boxplots of all light conditions at 18:00 and 02:00 at 690 dd, where outliers are indicated by red asterisks and the median is shown as a line inside each box. A and C) The two *nr1d4b* genes showed a similar expression pattern through circadian sampling. E and G) The genes *arntl2b* and *arntl2c* showed a similar trend but only *arntl2b* showed a decrease in expression at dark phase (02:00). B and D) For both *nr1d4b* genes, expression was lower at 02:00 compared to 18:00 in LDM and light-dark of red (LDR). Statistically significant differences were confirmed by conducting Wald test in DESeq2, where downregulation was significant in LDM for both genes and in LDR for *nr1d4b*.*1*. F and H) Validated by DESeq2, *arntl2c* was significantly upregulated during light phase (18:00) in all light conditions compared to continuous darkness (DD).

In the circadian series dataset with a light-dark cycle (14:10) of medium intensity of white light (LDM), 595 genes were shown to cycle at p < 0.05, 123 of these genes were cycling at p < 0.01 and 21 genes at p < 0.001 (S6 Table). Among the 595 cycling genes, 227, 165 and 203 genes cycled with a periodicity of 20 h, 24 h and 28 h, respectively. Pathway enrichment analyzes of these genes (both analyzing the genes based on periodicity (20 h, 24 h and 28 h) and all 595 genes together gave no overrepresented ontologies. In the list of genes (p < 0.05), two were circadian clock genes, *nr1d4b*.*2* (also referred to as *rev-erb*) (ENSSSAG00000067342) and *arntl2b* (also referred to as *bmal*) (ENSSSAG00000008440), that cycle with a periodicity of 28 h (Fig 2A and 2E). The expression profile of the Ss4R ohnologue *nr1d4b*.*1* (ENSSSAG00000081117) showed a similar expression pattern as *nr1d4b*.*2* (Fig 2C) while the ohnologue *arntl2c* (ENSSSAG00000009920) did not have a decrease in expression in dark as for *arntl2b* (Fig 2G). However, the expression level of *arntl2c* was 10-fold higher than *arntl2b* during the circadian sampling series. The peak of expression for both *nr1d4b* ohnologues were at 18:00, with expression levels being decreased during the dark phase (Fig 2A and 2C). For *arntl2b*, expression was lowest at 02:00 (Fig 2E). Boxplots of the expression levels in all light conditions at 18:00 and 02:00 (Fig 2B and 2D and 2F and 2H) were made to show the dynamics at constant conditions and between light- and dark phases. The plots show that for both *nr1d4b* ohnologues, expression decreased at 02:00 compared to 18:00 in LDM and light-dark of red (LDR) (Fig 2B and 2D). Differentially gene expression by DESeq2 showed that this downregulation was significant in LDM for both genes and in LDR for *nr1d4b*.*1*. DESeq2 analyzes also revealed that *arntl2c* was significantly upregulated during the light phase (18:00) in all light stimulations compared to continuous darkness (DD), as illustrated in Fig 2H.

### Transcriptomic changes under different light conditions

**Fig 3:**
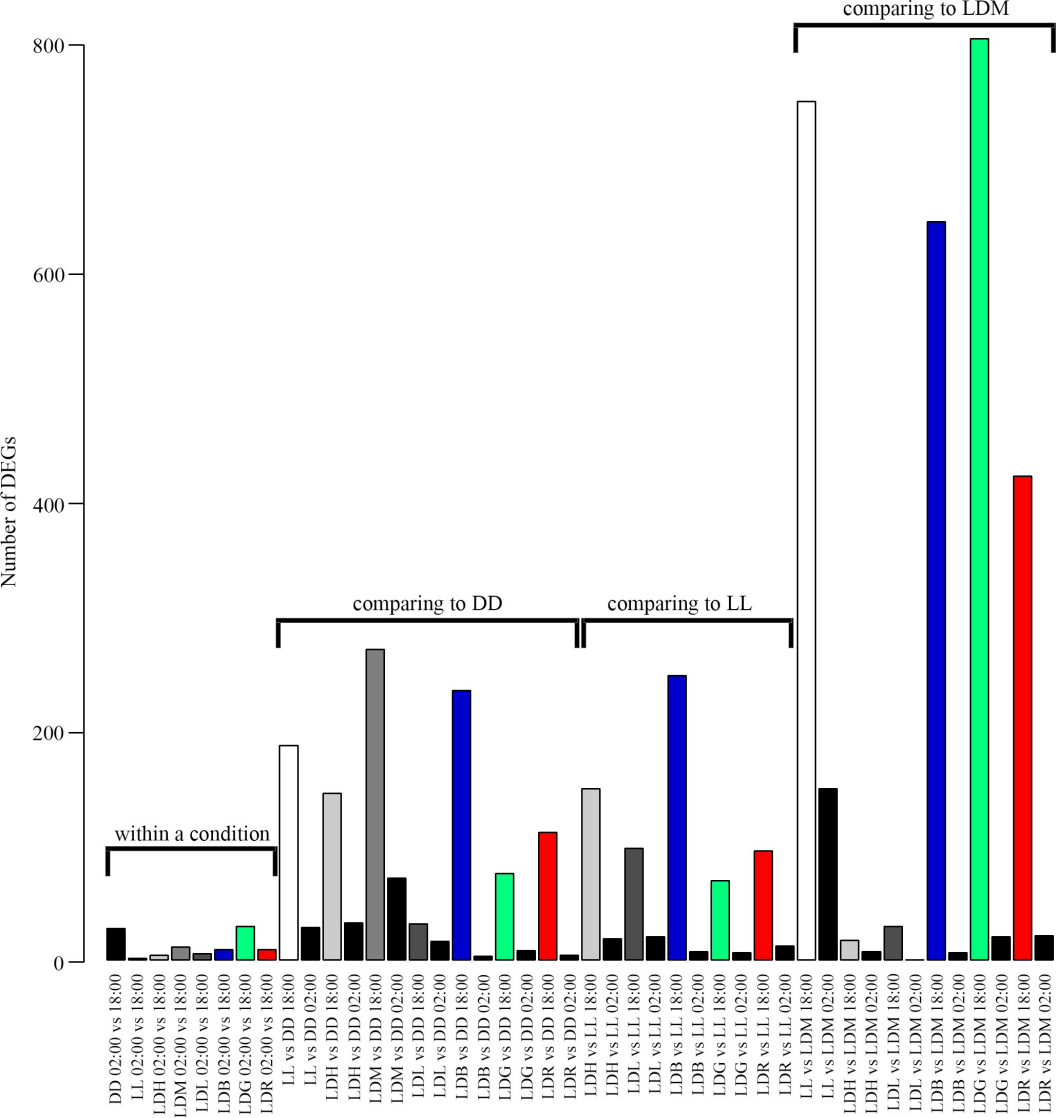
Bar chart comparing differentially expressed genes (DEGs). The bar chart shows the number of DEGs within a light condition comparing the expression at 18:00 and 02:00 (within a condition), between conditions both at 18:00 and 02:00 (comparing to continuous darkness, DD), (comparing to continuous white light, LL) and (comparing to light-dark (LD) of white light of medium intensity, LDM). Few genes were differentially expressed within a condition at 18:00 and 02:00. Comparing the different light conditions to DD, LL or LDM revealed more DEGs 18.00 then 02:00. The color code within a condition is DD (black), LL (white), LDH (light grey), LDM (medium grey), LDL (dark grey), LDB (blue), LDG (green), LDR (red). In comparing to DD, LL and LDM the color at 18:00 is reflecting the condition being examined (as for within a condition), and at 02:00 it is black.

**Fig 4:**
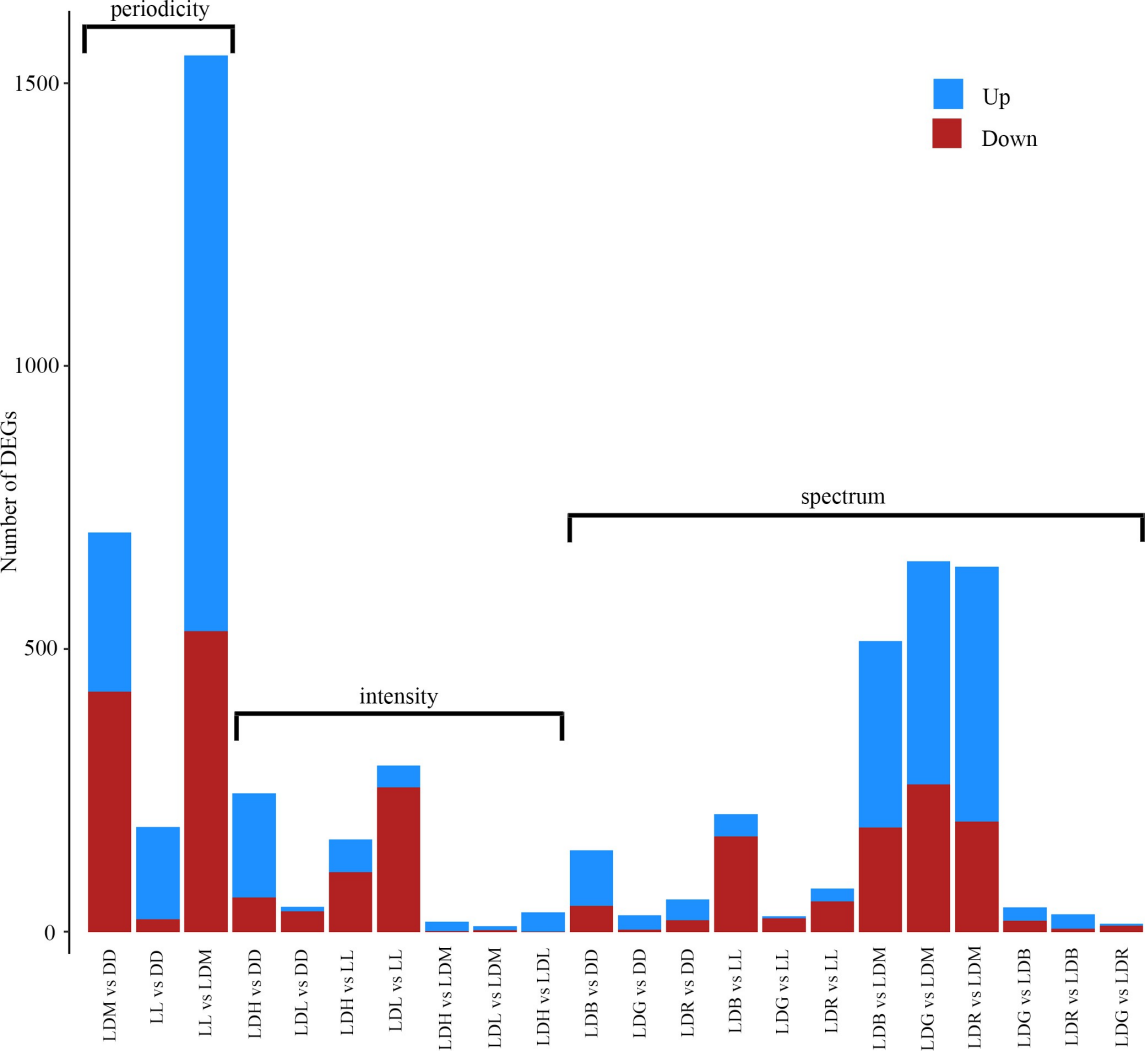
Bar chart comparing differentially expressed genes (DEGs) when 18:00 and 02:00 samples are combined. Many genes are differentially expressed when comparing constant conditions (DD or LL) to LDM. Different intensities of white light had few DEGs when compared to each other. Comparing different wavelengths of light (LDB, LDG and LDR) to LDM gave many DEGs while few DEGs were apparent compared to DD, LL or to each other. LL, LDB, LDG and LDR had the same intensity as LDM.

Differential gene expression analyzes by DESeq2 were conducted on the circadian sampling series and showed that not many genes were differentially expressed when comparing timepoints. Analyzes were done using different controls, 10:00 start, 18:00, 02:00 or an altering control (S7 Table), and all gave few differentially expressed genes (DEGs). DESeq2 was further used to examine differential gene expression between light (18:00) and dark phase (02:00) and the analyzes were conducted both within the same light condition and between light conditions comparing either to the continuous conditions of darkness (DD) or white light of medium intensity (LL), or to cycles of white light of medium intensity (LDM) (S7 Table and Fig 3). There were not many differentially expressed genes between 18:00 and 02:00 within a light condition before first feeding. However, comparing to DD, LL or LDM revealed that many genes were differentially up or downregulated between light conditions. Interestingly, there was a high number of DEGs during light phase (18:00). The number of DEGs was also higher when using LDM as control compared to DD and LL. Since there were few DEGs between 18:00 and 02:00 within a condition and in the circadian sampling series of LDM (S7 Table) the samples from 18:00 and 02:00 were combined, and Fig 4 shows a bar chart of the number of up and downregulated DEGs when the samples were combined, showing in general a higher number of DEGs when comparing to LDM than DD or LL. Comparing continuous conditions (DD and LL) to periodicity of white light (LDM) gave many DEGs, especially between continuous white light (LL) and periodicity of white light (LDM). Examination of the intensities of white light, revealed that only a few genes were differentially expressed between LDH (High) and LDM (Medium), LDL (Low) and LDM and LDH and LDL. In the different colors, the number of DEGs were high when comparing to LDM but not when alevins from different narrow bandwidth light (LDB (Blue), LDG (Green), LDR (Red)) were compared to each other.

### Gene ontology and photoperiod

**Fig 5:**
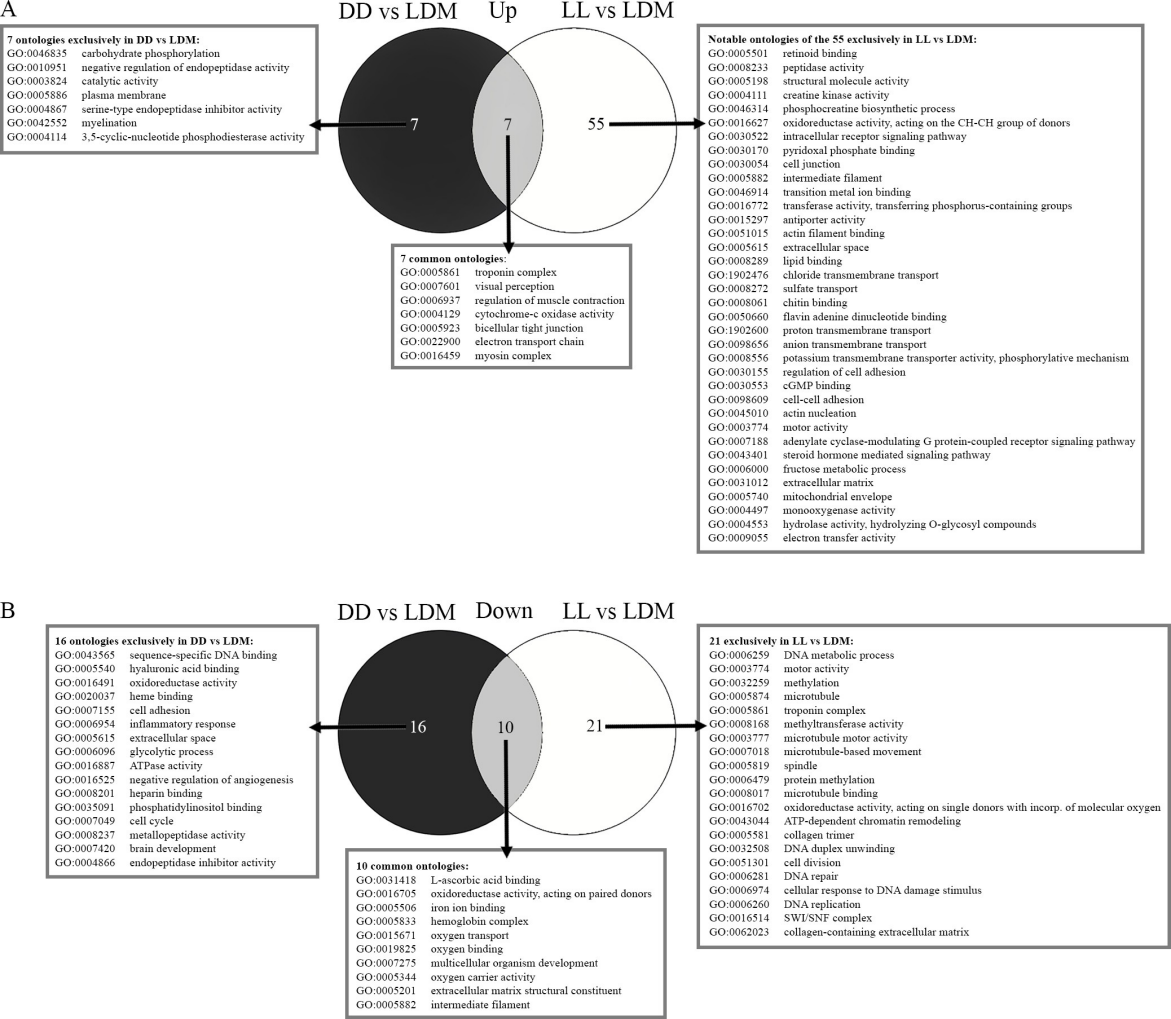
Venn diagrams of Gene Ontologies (GO) comparing different photoperiods. A) Upregulated GO terms comparing a constant lighting environment (DD or LL) to LDM revealed 7 common ontologies. In LL, 55 GO terms were upregulated (see S8 Table for a complete list). B) Downregulated GO terms comparing constant environment (DD or LL) to LDM, 10 common ontologies were found.

Analyzing the Gene Ontology (GO) terms of periodicity, when comparing constant conditions (DD and LL) to LDM, revealed that seven GO terms were upregulated in constant conditions (Fig 5A and S8 Table), namely those associated with “visual perception” and “regulation of muscle contraction”. In DD, GO terms related to endopeptidase activity (“negative regulation of endopeptidase activity” and “serine-type endopeptidase inhibitor activity”) and “catalytic activity” were among those upregulated compared to LDM. In LL, 55 GO terms were exclusively upregulated compared to LDM and in Fig 5A the notable ontologies are listed, see full list in S8 Table. Several of the upregulated GO terms in LL were related to metabolic processes such as “fructose metabolic process”, “phosphocreatine biosynthesis”, “fatty acid metabolism” and related terms of peptidase activity. GO terms associated with transmembrane transport, cellular anatomy such as “intermediate filament” and “extracellular matrix” were also upregulated in LL. Interestingly in LL, the terms “nuclear receptor activity” and “steroid hormone receptor activity” are enriched by circadian clock genes. The analyzes showed that 10 GO terms were downregulated under constant conditions (LL and DD) compared to LDM (Fig 5B and S8 Table). The GO terms “hemoglobin complex”, “oxygen binding”, “oxygen carrier activity” and “oxygen transport” were all enriched by genes coding for subunits of hemoglobin and “L-ascorbic acid binding”, “oxidoreductase activity”, “iron ion binding” are enriched by hydroxylase genes. Strikingly, “multicellular organism development” was downregulated in constant conditions. In DD biological processes such as “brain development” and “cell cycle” were down regulated, as well as metabolic processes such as the “glycolytic process” and “peptidase activity”. In LL, GO terms downregulated included “cell division”, “DNA replication”, “DNA duplex unwinding”, “methylation”, “chromatin remodeling”, “DNA repair” and the “cellular response to DNA damage”. Several GO terms related to microtubules including “microtubule motor activity” were also downregulated in LL. Counterintuitively, some GO terms in LL were listed as being both upregulated and downregulated, such as “troponin complex”, “motor activity”, “intermediate filament”, “collagen-containing extracellular matrix” and “iron ion binding”, however they were enriched by different genes.

### Gene ontology and different wavelengths of light

**Fig 6:**
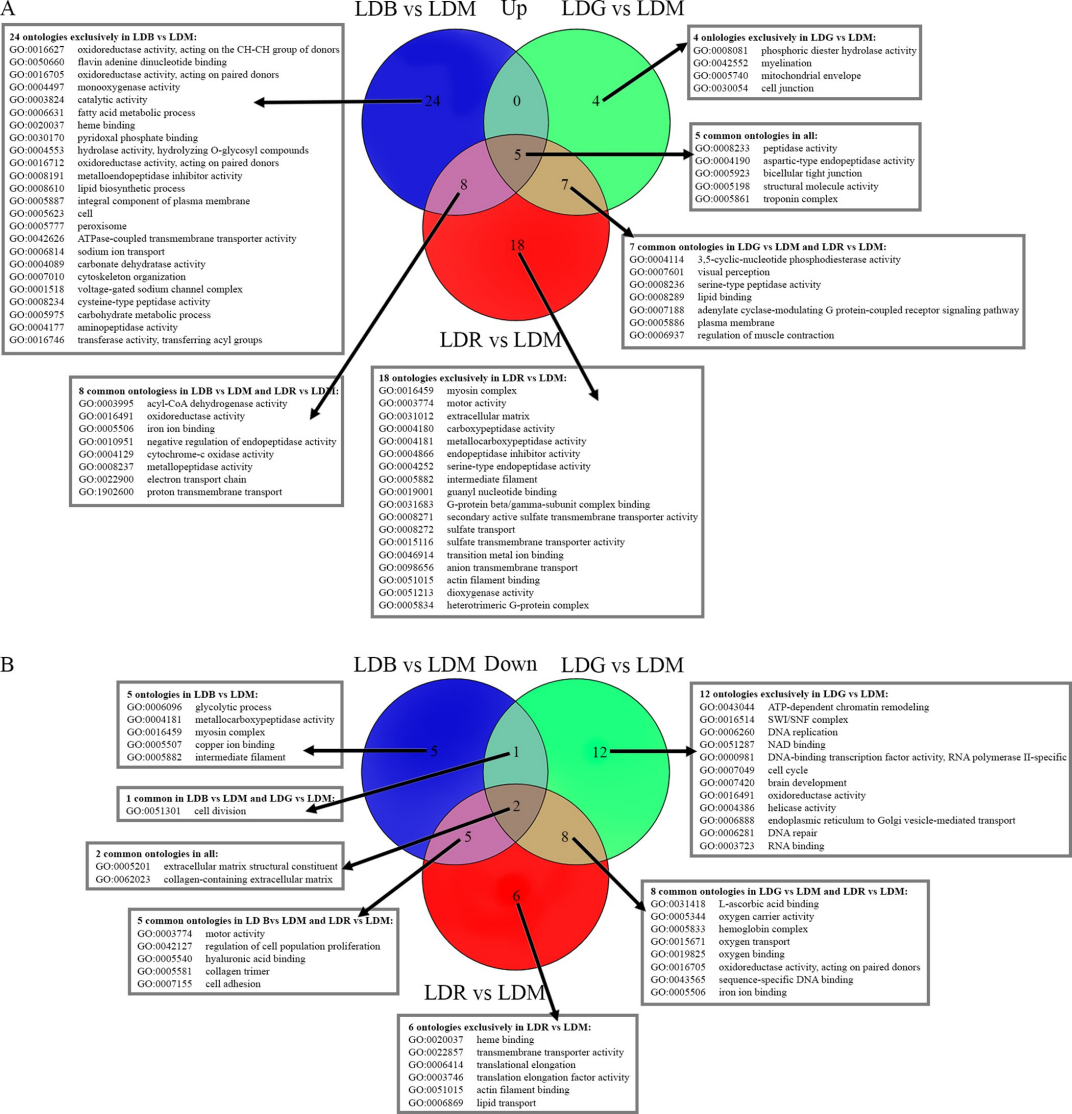
Venn diagrams of Gene Ontologies (GO) comparing wavelengths used during the light phase of the LD cycle. A) Upregulated GO terms when comparing blue, green and red light to white light (LDM), where five ontologies were common in all lighting conditions. B) Downregulated GO terms comparing blue, green or red light to white light (LDM), where two ontologies were found to be common despite the use of different wavelengths.

Alevins reared in LD cycles containing blue, green, or red light gave five common upregulated GO terms in all three color-enriched conditions compared to white light (LDM) (Fig 6A and S8 Table). There were eight common terms between LDB and LDR (e.g. “cytochrome-c oxidase activity” and “acyl-CoA dehydrogenase activity”) and seven common terms between LDG and LDR compared to LDM (e.g. “peptidase activity”, “muscle contraction” and “visual perception”). Some GO terms were exclusively upregulated in LDB (e.g. “fatty acid metabolic” and “lipid biosynthetic processes”) and in LDG (e.g. “phosphoric diester hydrolase activity” related to photoreception). In LDR, several GO terms associated with peptidase activity, sulfate transport, the G-protein complex and cellular anatomy such as “intermediate filament” were upregulated. Two downregulated GO terms associated with extracellular matrix were common to all spectral experiments that differed in the wavelengths being emitted during the light phase, and several GO terms were linked to extracellular matrix in LDB and LDR (Fig 6B and S8 Table). In LDG and LDR, GO terms linked to oxygen processes such as “oxygen binding”, “oxygen carrier activity” and “oxygen transport” were downregulated. In LDB and LDG, the GO term “cell division” was also downregulated. One GO term relating to glycolysis was exclusively downregulated in LDB, whereas several GO terms associated with DNA replication, transcription and repair were downregulated in LDG, as well as the terms “brain development” and “cell cycle”. In LDR, GO terms related to transport activity and transcriptional elongation were also downregulated.

### The KEGG pathway “phototransduction”

**Fig 7:**
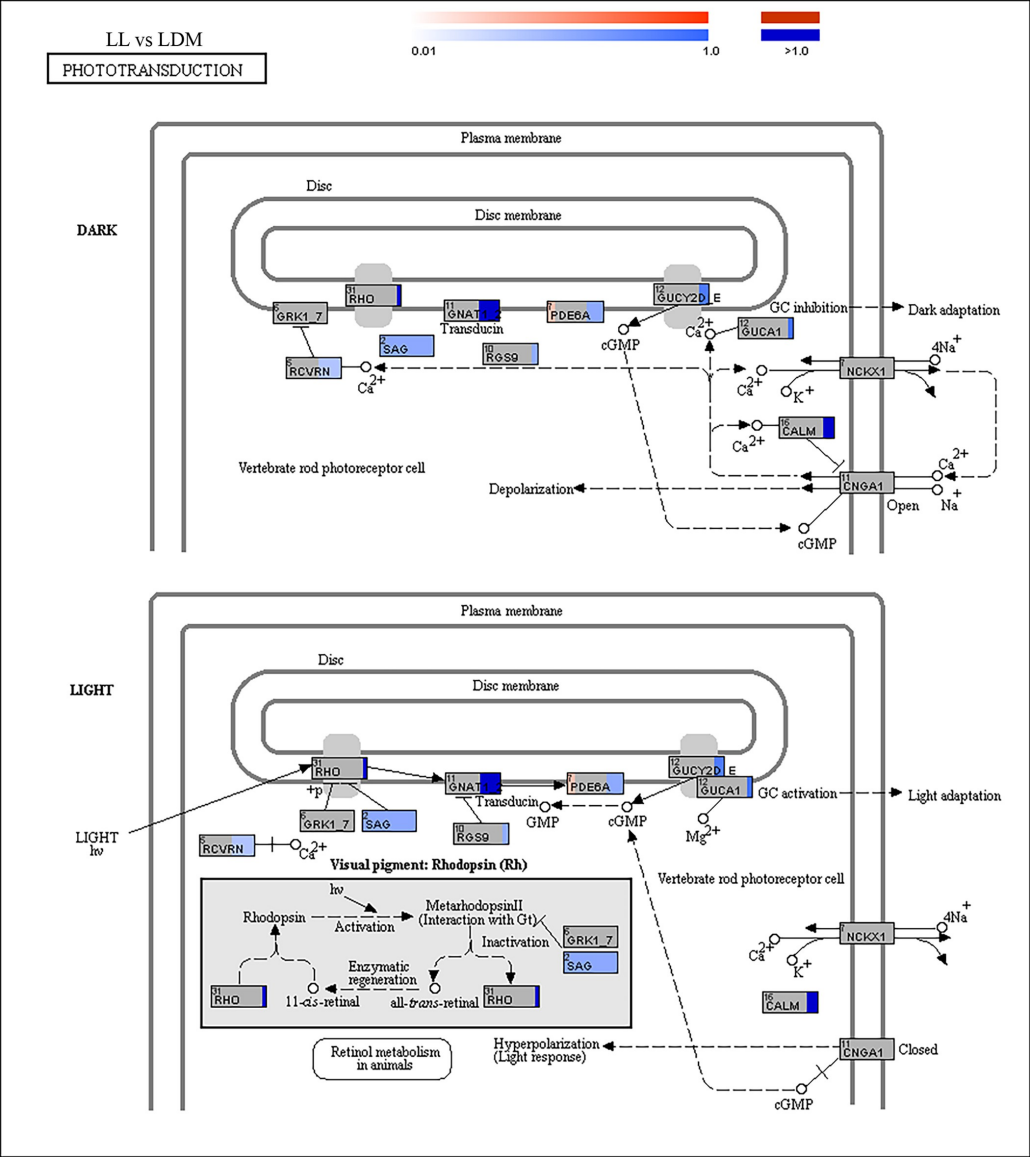
Differentially expressed genes between continuous white light (LL) and periodicity of white light (LDM) apparent in the Kyoto Encyclopedia of Genes and Genomes (KEGG) pathway “phototransduction”. The scale indicates the logfold2 change and the color code indicate upregulated (blue) and downregulated (red) genes. There were 20 genes apparent in the phototransduction pathway.

Visual perception and G-protein related ontologies were apparent in the pathway enrichment analyzes comparing periodicity and different wavelengths to LDM and further detailed analyzes were done by generating KEGG pathway “phototransduction” figures (Figs 7 and S6). There are 20 and 16 differentially expressed genes in the phototransduction cascade comparing alevins raised in LL and DD to alevins in a periodicity of white light (LDM), respectively (Figs 7 and S6A). Comparing LDM to different wavelengths of light, gave four genes in LDB (S6B Fig) and 13 genes in both LDG (S6C Fig) and LDR (S6D Fig). As for visual perception and G-protein related ontologies most of the genes are upregulated comparing to LDM.

## Discussion

To determine how light quality through the axes of periodicity, intensity and spectrum impacts development through transcriptome activation, a four-month light exposure experiment was performed with Atlantic salmon embryos from fertilization to the alevin stage. The development of nonvisual photoreception and clock genes were also included to obtain an overview of photoreceptive capacity and cycling regulators. Resultant RNA sequencing results provided a unique insight into the impact of light on the transcriptome.

### Transcriptome development, nonvisual photoreceptor and circadian clock ontogeny

In recent years, a high-quality genome assembly for Atlantic salmon has been published together with 46,598 genes identified by annotation of gene structures using RNA sequencing results and expressed sequence tags [12]. The present study showed that many of these genes are expressed at early developmental stages, with 34,414 genes expressed at 255 dd when eye pigmentation occurs. However, many genes were differentially expressed when comparing the developing embryo to pre-first feeding alevins, noting that upregulated GO terms were mainly related to development. The ontologies are consistent with developmental changes from an embryo at the eye pigmentation stage to an alevin that is ready for first feeding. For example, many ontologies are related to the mitotic cell cycle, RNA processing and transcription, which reflect the growing embryo and alevin throughout the experiment. The ontology “brain development” was apparent at all stages compared to 690 dd. Among the enriched genes were homeobox genes within the *six3* class and the class III POU-domain, known to be expressed in the developing zebrafish brain [39,40]. The enrichment analyzes also showed that several biological processes such as the “immune system”, “visual perception” and “regulation of muscle contraction” were apparent first just prior to first feeding. These results are consistent with a free swimming first feeding ready alevin that requires a functional visual system to maximize the capture of prey.

Analyzes of nonvisual photoreceptive capacity showed that several nonvisual opsins were already present at 255 dd suggesting that nonvisual photoreception appears before that mediated by the visual system. Early expression of nonvisual opsins is in accordance with previous findings in Atlantic halibut [17,26] and zebrafish [19,20]. Approximately a third of the repertoire of nonvisual opsin genes were expressed early, with the rest being activated towards first feeding. Circadian clock genes were all expressed at 255 dd and increased in expression towards first feeding. As reported for clock genes [41], the number of nonvisual opsin Ss4R pairs and singletons (14 pairs and 14 singletons) are consistent with analyzes of salmonids genomes, demonstrating that 55% and 48% of the Ss4R duplicates are retained with two functional copies in Atlantic salmon and rainbow trout, respectively [12,42]. The differential expression of nonvisual opsin ohnologues shown in the present study, supports previously published data showing that 45% of well-defined expressed Ss4R pairs had signs of diverged expression across 15 tissues in salmon [12]. Interestingly, in rainbow trout divergent tissue expression patterns between ohnologues are specifically enriched by genes related to photoreception, eye development and visual perception [42]. Importantly, the present findings confirm the early photoreceptive capacity of salmon embryos and indicates that light cues applied in this study from fertilization to first feeding were perceived.

### The circadian clock before first feeding

The present study included a circadian sampling series that showed that 595 genes were cycling (p < 0.05) before first feeding, however, no GO terms were overrepresented when performing pathway enrichment analyzes. Interestingly, the two clock genes, *nr1d4b*.*2* and *arntl2b*, cycled with a period of 28 h, which is different to normal day-night conditions where the clock phase is entrained to a 24-hours period [43]. The reason for an extended cycling period in these two genes is not clear, but it might be because the circadian clock is not fully established before first feeding. By comparison, 487 cycling genes (p < 0.05) with periods of 20, 24 or 28 h were identified in a model cnidarian species. In this study, whole animals were used in a similar circadian sampling series under full spectrum light and JTK_CYCLE script with shortened and extended period lengths included in the analyzes [44]. Recently, a circadian sampling series in Atlantic salmon parr brain has revealed 2,864 rhythmically expressed genes (p < 0.001) These included 1,215 genes with a circadian expression pattern, among them 11 clock genes with a 24 hours cycling period [38], supporting the presence of a well-established circadian clock. Notably, the two clock genes highlighted in the presented study (i.e. *nr1d4b*.*2* and *arntl2b*) are not among the 11 cycling clock genes in salmon parr. In zebrafish, a microarray study providing a whole genome transcriptional profile revealed 2,856 circadian cycling genes in 5 days post fertilization larvae [45]. Annotation of biological function showed that resultant GO terms were related to light, abiotic stimuli and transporter function. The most significant enriched KEGG pathway corresponded to circadian rhythms [45]. In addition, the photoresponsive transcriptome of zebrafish has been studied by microarray in larvae reared in darkness and exposed to light stimulations. Upregulated GO terms after light exposure included those related to circadian rhythms, the stress response and DNA repair [35]. Interestingly, *LON peptidase N-terminal domain ring finger 1* (*lonrf1*) was strongly upregulated after light induction in zebrafish and is suggested to be involved in DNA repair [35]. In the present study, *lonrf1* was one of the 595 cycling genes and the gene family that was upregulated in several DESeq2 analyzes when comparing different light conditions to DD.

Another microarray study performed in gilthead sea bream larvae at first feeding revealed 2,229 differentially expressed genes throughout the day. The analyzes demonstrated a coordinated daily development of cellular and metabolic processes, with circadian variations in the transcriptome resembling that of cell cycle progression. The circadian clock in start feed larvae was shown to be highly synchronized, supported by coordinated peaks in main clock gene expression at different timepoints [34]. Conversely, this current study was conducted before first feeding when salmon alevins still rely on their yolk. Only two circadian clock genes were rhythmic; one gene belonged to the core clock transcriptional activator *bmal* family [46] and the other gene to the secondary loop *rev-erb* family, know to repress *bmal* transcription [47]. In agreement with results obtained in gilthead sea bream larvae, members of the *bmal* and *rev-erb* families exhibited similar expression patterns, with a decreased expression during the dark period [34]. However, a peak in the expression of the *rev-erb* family gene at 18:00 was found in this study, not seen for *bmal*. Interestingly, in mouse it has been shown that the CLOCK/BMAL1 heterodimer drives the rhythmic transcription of *REV-ERBα*, which then feeds back to repress *Bmal1* gene transcription [48]. The results presented here showed that *rev-erb* family ohnologues have similar expression patterns, even though only one of the genes appeared in JTK_CYCLE. For the *bmal* family ohnologues, expression levels differed by 10-fold and only one gene cycled, suggesting that the whole genome duplication event in salmonids may have led clock genes to diversify into non-circadian functions [41]. Expression of *rev-erb* family genes were downregulated at night in LDM and LDR groups; this was significant in LDM for both genes and for one of the genes in LDR. However, no such downregulation was observed in either LDB or LDG groups, suggesting that expression of these genes is regulated by long wavelength light. Overall, clock genes in Atlantic salmon showed an unexpected lack of cycling at this stage of development.

### Impact of photoperiodicity, intensity and spectrum on the transcriptome

Only a few differentially expressed genes were found between day and night (18:00 and 02:00) within a specific lighting condition: in the circadian sampling series in LDM, only a few DEGs were found between all timepoints analyzed, which suggests that photoperiodicity does not have a large influence on gene expression in salmon before first feeding. These results contrast with previously published data from gilthead sea bream larvae at first feeding stage, in which 2,229 DEGs were identified throughout the day (e.g. 633 DEGs when comparing 18:00 and 03:00) [34]. However, when comparing the different light conditions to DD, LL or LDM, many DEGs, especially at daytime, were identified. Surprisingly, the numbers of DEGs were greater at daytime than at night, when compared to DD, LL or LDM, which contrasted with the few DEGs found between day and night within a lighting condition. This suggests that the effect of different light stimulations at daytime is not maintained at night. Further, the numbers of DEGs were highest for LL and different light spectra compared to LDM, while fewer DEGs were identified when comparing LDB, LDG and LDR to each other. When analyzing different light intensities, most DEGs were seen compared to darkness. Comparisons between the intensities gave few DEGs, suggesting that the difference in photons flux (0.01–1.0 W/m^2^) between LDL, LDM and LDH was not enough to elicit differential expression. Comparing LDL and DD gave fewer DEGs compared to higher intensities; however, the light intensity threshold in Atlantic salmon pineal is between 3.8 x 10^−4^ W/m^2^ and 3.8 x 10^−5^ W/m^2^. Also, it has been shown that light intensity of 2,6 x 10^−3^ W/m^2^ is enough to reduce the melatonin level by 66% compared to darkness [49] and LDL was well above this range. The number of DEGs are not cumulative when comparing low to high intensities of white light compared to darkness, with highest number of DEGs in LDM. The low number of DEGs also suggests that the light intensities tested in the present study were within a range that did not cause major stress. This is consistent with previous studies that showed that high intensities of blue light (2.7 W/m^2^) displayed increased plasma cortisol and glucose levels within 3 h, both indicative of a stress response, while lower intensities of blue light (0.2 and 0.75 W/m^2^) and white light (2.1 W/m^2^) did not [50].

### Up- and downregulation in constant and narrow bandwidth light conditions

#### Photoreception

Pathway enrichment analyzes showed that photoperiodicity influenced the transcriptome, with several GO terms being up- or downregulated in constant conditions. Among the upregulated GO terms, “visual perception” was apparent in both DD and LL, with some common enriched genes. Alevins reared in DD, however, showed upregulation of rod opsin, which is important for dimlight vision, while cone opsins responsible for bright daylight vision [51] were upregulated in LL. Cyclic nucleotide-gated cation channel beta-1, involved in the regulation of ion flow into the rod photoreceptor outer segment [52,53], was apparent in darkness, with upregulation in the KEGG pathway “phototransduction” of DD compared to LDM. The KEGG pathway “phototransduction” figures of LL and DD compared to LDM also showed that most enriched genes are upregulated in constant conditions compared to white light periodicity. Photoreception is also influenced by narrow bandwidth light and in contrast to LDB, LDG and LDR shared upregulated ontologies related to photoreception. As in DD, rod opsin was enriched in LDR in the ontology “visual perception”. The alpha subunit of the guanine nucleotide-binding protein G, which is part of the transducin trimer that mediates between the visual photopigment and the effector enzyme in phototransduction (reviewed in [54]), was enriched in G-protein related ontologies in LDG and LDR. In LDG, the terms phosphoric diester hydrolase activity and 3,5-cyclic-nucleotide phosphodiesterase activity contained the beta subunit of cGMP- phosphodiesterase, that is the rod specific subunit of the effector enzyme [54]. These results indicate an increased activity of rod photoreceptors in green and red light compared to white light. Interestingly, in LDG under phosphoric diester hydrolase activity, the effector enzyme of phototransduction that is typical of rhabdomeric photoreceptors, the phospholipase enzyme, was enriched, indicating increased melanopsin-induced phototransduction in LDG [55]. The KEGG pathway “phototransduction” for LDB, LDG and LDR compared to LDM showed that few genes were apparent in LDB, but several genes are upregulated in LDG and LDR. These results indicate that a broad-spectrum white light and short wavelengths of (specifically blue light) lead to similar photoreceptive responses.

#### Development

The analyzes revealed that developmental processes are downregulated in different light conditions. The ontology “multicellular organism development” was downregulated in DD and LL and several of the enriched genes are homeobox genes, belonging to transcription factors families important for eye development such as *pax6*, *otx2*, *rx1* and *rax2* [56–59], suggesting that eye development may be affected in constant conditions. In DD, LDG and LDR, “sequence specific DNA binding” was enriched by genes belonging to the same transcription factor families. Further, in DD and LDG, the homeobox gene *six3* thought to have essential roles in defining eye primordia in zebrafish and shown to be expressed in eye and rostral brain during later stages [39], was among the enriched genes in the downregulated ontology “brain development”. The same homeobox gene was also enriched in “DNA-binding transcription factor activity” in LDG. Taken together, these results show that darkness and long wavelengths of light lead to a downregulation of transcription factors important for eye and brain development when compared to broad spectrum white light.

#### Cell cycle

In zebrafish, light has been shown to regulate the cell cycle by revealing that LD cycles cause different cell-types to enter the S phase towards the end of the day and that larvae raised in DD only have a low level of arrhythmic cells in the S phase [60]. Here, the ontology “cell cycle” was downregulated in DD and LL showed a downregulation of the terms “cell division”, “DNA replication” and “chromatin remodeling”, suggesting that several phases of the cell cycle were influenced in alevins exposed to constant conditions. These results may indicate that a lack of photoperiod influences the cell cycle as shown in zebrafish. Notably, in “DNA replication” and “DNA metabolic process”, proliferating cell nuclear antigen, known to play a key role in DNA repair, DNA methylation, chromatin remodeling and cell cycle regulation (reviewed in [61]), was apparent. In narrow bandwidth light, both LDB and LDG had downregulation of the term “cell division” and were enriched by the same genes, e.g. condensin which has an essential role in mitotic chromosome assembly and segregation [62]. Interestingly, in LDG the ontologies “cell cycle”, “DNA replication”, “chromatin remodeling” and “helicase activity” were downregulated, suggesting that different phases of the cell cycle were also influenced by green light.

#### DNA repair

The terms “DNA repair” and “cellular response to DNA damage stimulus” were downregulated in LL, suggesting that alevins in LL experience less DNA repair. Several of the enriched genes were apparent in the KEGG pathway “base excision repair” for Atlantic salmon, which is a repair mechanism known to correct small base lesions, resulted from deamination, oxidation, or methylation which can be caused by spontaneous decay of DNA, environmental chemicals, radiation, or treatment with cytostatic drugs (reviewed in [63]). By comparison, “DNA repair” was upregulated in the light responsive transcriptome of zebrafish larvae previously reared in darkness and the enriched genes indicate that cells are prepared for UV-induced damage repair [35]. “DNA repair” was also downregulated in LDG and together with “NAD binding” the enriched genes suggest downregulation of base excision repair as seen in LL.

#### Nuclear receptor activity

Comparing LL to LDM, the GO terms “nuclear receptor activity” and “steroid hormone receptor activity” were enriched with circadian clock genes. These terms showed an upregulation of genes in the *rev-erb* and *ror* families which are nuclear receptors repressing and activating *bmal* transcription, the core clock transcriptional activator, respectively [47]. The transcription of *bmal* is a result of competition between the *rev-erb*s and *ror*s, binding to retinoic acid–related orphan receptor response elements in the *bmal* promoter [48,64]. However, the significance in upregulation for both of these competing nuclear receptors in LL remains unclear.

#### Peptidase activity

Several upregulated GO terms in DD and LL are related to peptidase activity, which catalyzes the hydrolysis of a peptide bond. In DD, both up- and downregulated terms were associated with decreased peptidase activity, while upregulated terms in LL were mostly related to an increased activity of peptidases. Proteolytic enzymes from the pancreas, especially trypsin and chymotrypsin that hydrolyze peptide bond within proteins, are considered to be important during larval stages of fish [65] and in LL the ontologies were enriched by trypsin and chymotrypsin. The GO terms “peptidase activity” and “aspartic-type endopeptidase activity” were upregulated in all light spectra, with LDB and LDR having several other upregulated ontologies related to peptidase activity, mostly associated with increased activity. Together, these results indicate that in darkness there was decreased hydrolysis of peptide bonds, while both continuous white light and narrow bandwidth light had increased hydrolysis compared to LDM.

#### Metabolism

The upregulated ontologies “carbohydrate phosphorylation” and “catalytic activity” in DD and several ontologies related to fructose metabolic process in LL are enriched by fructose-2,6-bisphosphatase. Upregulation of fructose-2,6-bisphosphatase indicates a breakdown of glucose by the glycolytic process [66]. However, the term “glycolytic process” was downregulated in DD and in LDB, with the enriched genes being associated with different steps of glycolysis. In LL, LDB and LDR, the term “acyl-CoA dehydrogenase activity” was enriched by genes apparent in the KEGG pathway of “fatty acid degradation” in Atlantic salmon and in LL the “phosphocreatine biosynthesis” was upregulated. In LDB and LDR the common term “oxidoreductase activity” was enriched by genes both related to fatty acid degradation and cholesterol metabolism (e.g. cholesterol 7-alpha-monooxygenase). In addition, LDB had several additional terms associated with fatty acid metabolism. Taken together, these results indicate an increased availability or use of energy in alevins raised in LL, LDB and LDR.

#### Oxygen homeostasis

Several of the common downregulated GO terms in DD ad LL were enriched by genes coding for hemoglobin subunits and three ontologies (“ion-ion binding”, “oxidoreductase activity” and “L-ascorbic acid binding”) were enriched by genes related to hydroxylase activity. Among the enriched genes, there were prolyl hydroxylase domain-containing genes, which code for proteins that catalyze the hydroxylation of proline residues of the hypoxia inducible factor (hif), central in oxygen homeostasis e.g. during embryonic development [67,68]. The hif protein is hydroxylated during normal oxygen levels, resulting in proteasome degradation. However, under hypoxic conditions, this factor accumulates, resulting in the expression of target genes involved in cellular and systemic responses to hypoxia [67,68]. As for constant conditions, LDG and LDR exhibited the same downregulated ontologies enriched by genes coding for hemoglobin subunits and those associated with hydroxylase activity. A downregulation of genes responsible for the catalysis of hydroxylation in suggests that there was an increased oxygen consumption in constant conditions, LDG and LDR, when compared to LDM.

#### Muscle contraction

In constant conditions, “regulation of muscle contraction”, “troponin complex” and “myosin complex” were enriched by various subunits of the troponin and myosin gene families, respectively, which related to both skeletal and cardiac muscle contraction [reviewed in [69]). The term “troponin complex” was also upregulated in all spectra and enriched by many of the same genes as for constant conditions. In addition, LDG and LDR shared the term regulation of muscle contraction and LDR exhibited several GO terms enriched by genes of the myosin family. The upregulation of these terms indicates an increased regulation of muscle contraction and/or muscle activity in these light conditions compared to LDM, which may indicate an increase in movement of alevins under these conditions. Further, the gene ontology “cytochrome-c oxidase activity” is upregulated in constant conditions, and in LDB and LDR, and is enriched by different subunits of cytochrome-c oxidase, that is the terminal enzyme of the mitochondrial respiratory chain that results in synthesis of adenosine-5’-triphosphate, the immediate energy source for multiple biological processes reviewed in [70]). These data suggest an increased availability or use of energy.

## Conclusion

Atlantic salmon is a promising model for studying the impact of light on development since its life cycle is tightly controlled by seasonal changes in photoperiod. In this study, the periodicity, intensity and spectrum of light were analyzed at a stage when the embryo still engages in endogenous feeding from the yolk. Detailed transcriptome analyzes showed that nonvisual opsins are expressed early in development, implying the presence of early photoreceptive capabilities that can be used to modulate biological processes. Although the clock system is present early in salmon, it seems to have very limited clock cycling activity prior to first feeding. The divergence of transcriptome profiles between light conditions may represent a direct modulation of the transcriptome by nonvisual photoreceptors, and not through direct photoreceptive control of the clock system. Further, a change in photoperiod and spectrum had a greater influence on the transcriptome than different intensities of white light, showing an overrepresentation of genes in pathways related to development, photoreception, the circadian clock, DNA repair and metabolism. This may represent biological processes that are under direct light control and independent of the cycling clock system.

## Materials and methods

### Ethics statement

All experiments followed the local animal care guidelines and were done in facilities that were given approval by the Norwegian Food Safety Authority (VSID2135). According to the Norwegian Regulation on Animal Experimentation (FOR-2015-06-18-761), fertilized eggs and alevins before exogenous feeding are exempted by the regulation and thus do not require specific approval of the experimental protocol. However, the experiment was done in strict accordance with the Norwegian Animal Welfare Act (LOV-2009-06-19-97) and complied with the ARRIVE (Animal Research: Reporting of In Vivo Experiments) guidelines [71].

### Animals

The study was performed on full siblings, by obtaining eggs (approx. 15 000) and sperm from one female and one male of Atlantic salmon (*Salmo salar*) from Mowi, Tveitevågen, Norway. Fertilization took place at an approved laboratory facility at the High Technology Center, University of Bergen, Norway, where all light stimulations were conducted from fertilization to start of feeding.

### Light experiments

Eggs and alevins were incubated under different light conditions using state-of-art light-emitting diode (LED) technology (Signify, The Netherlands), applying four different LEDs. The experimental setup and spectral properties of the LEDs are shown in S1 Fig and Table 1. A broad-spectrum light was defined as white light and three narrow bandwidth lights were specified as blue, green and red (see S1 Fig and Table 1 for range and λ_max_ values). Eight different light conditions, with triplicated incubators for each condition, were applied from fertilization to start feeding including continuous white light (LL); continuous darkness (DD); continuous light and dark (LD) periods using white light with three different intensities (High (LDH), Medium (LDM), Low (LDL)) and colored LD conditions using the narrow bandwidth LEDs (Blue (LDB), Green (LDG) and Red (LDR)). The LEDs were adjustable, where photon flux was measured and adjusted by a spectroradiometer (Ramses ACC-VIS, TriOS, Germany), setting LL, LDM, LDB, LDG, LDR to an experimental medium intensity (Table 1). The intensities were chosen based on light sensitivity thresholds [49] and to avoid high intensities that might induce stress responses (51). The LD cycle was 14L:10D, where lights were switch on and off at 08:00 and 22:00, respectively, with an incremental transition period of 30 min when changing between lighting periods; this regime provided an extended period of light exposure, but also included a substantial dark period. The temperature has a major impact on development, so the temperature was equalized in all tanks, including individual temperature measurements every 10 minutes. The average temperature during the experiment from fertilization to first feeding (121 days) was 6 ± 0,5°C. At the last sampling (day 113), before first feeding, the age of the alevins in the different tanks was 690 day degrees (dd), referring to the average temperature in all tanks per day multiplied by the number of days. Comparing the age in dd between tanks at the last sampling point, the difference was minimal, 690 ± 6 dd.

**Table 1:** Properties of the light-emitting diodes (LEDs). The intensities of the LEDs were adjusted by measurements by a spectroradiometer. For the white light, three intensities were used high (1.0 W/m^2^), medium (0.1 W/m^2^) and low (0.01 W/m^2^) and the photon flux was adjusted, accordingly. For different spectral experiments, green was set as 0.1 W/m^2^ and blue and red were adjusted to the same photon flux. (μE/m^2^/s = μmol/m^2^/s).

LED	Periodicity	Color	Intensity	Range (nm)	λmax (nm)	μE/m^2^/s	W/m^2^
1	Light:Light (LL)	White	Medium	414–781	610	0.4925	0.1004
2	Light:Dark (LD)	White	High	414–781	610	4.9035	1.0002
3	Light:Dark (LD)	White	Medium	414–781	610	0.4932	0.1004
4	Light:Dark (LD)	White	Low	414–781	610	0.0507	0.0103
5	Light:Dark (LD)	Blue	Medium	374–510	450	0.4504	0.1199
6	Light:Dark (LD)	Green	Medium	474–631	535	0.4509	0.1009
7	Light:Dark (LD)	Red	Medium	594–698	660	0.4505	0.0824

### RNA extraction

Eggs and alevins (n = 6–8 per stage), incubated in LDM were snap frozen directly in liquid nitrogen at different developmental stages (255 dd (40 days), 379 dd (60 days), 555 dd (90 days) and 690 dd (113 days)) that correspond approximately to when eye pigmentation occurs, before hatching, after hatching and before first feeding, respectively. At 690 dd, a circadian study was done with alevins reared under LDM conditions sampled (n = 4) at 4 h intervals during 24 h (10:00 start, 14:00, 18:00, 22:00, 02:00, 06:00 and 10:00 end, corresponding to zeitgeber time (ZT) ZT2 start, ZT6, ZT10, ZT14, ZT18, ZT22, ZT2 end). For other light conditions (LL, DD, LDH, LDL, LDB, LDG, LDR), sampling (n = 4) was conducted during simulated day (18:00, ZT6) and night (02:00, ZT18) phases (S1 Fig). In DD and at night the sampling was done using dim red light, and at all sampling points the eggs and alevins were snap frozen directly after taking them out of the incubators to accomplish a short sampling period. RNAlater ICE (ThermoFisher Scientific, Waltham, MA, USA) (-80°C) was added and the embryos and larvae were immersed at -20°C least 48 h before isolating total RNA by using TRI reagent (Sigma, St. Louis, MO, USA) according to the manufacturer’s instructions. Total RNA was DNase I treated by using the TURBO DNA-free Kit (ThermoScientific, Waltham, MA, USA). RNA integrity was monitored using Agilent2100Bioanalyzer (Agilent Technologies, SantaClara, CA, USA) revealing RNA integrity number (RIN) of samples valued between 8.8–10.

### RNA sequencing

In total, 102 RNA samples were submitted to the Genomics Core Facility at the University of Bergen for RNA sequencing. Each sample (400 ng) was processed and sequenced using the Illumina TruSeq Stranded mRNA Sample Preparation Kits according to the Illumina TruSeq Stranded mRNA Sample Preparation Guide on the Illumina HiSeq 4000 platform (Illumina, Inc., San Diego, CA, USA). Sequencing generated an average of 38 million 75 bp paired end reads per sample, which is within the recommendations from Illumina (https://emea.support.illumina.com/bulletins/2017/04/considerations-for-rna-seq-read-length-and-coverage-.html). All RNA sequencing data has been deposited to the European Nucleotide Archive Accession number PRJEB51921.

### RNA sequencing analyzes

The RNA sequencing results were trimmed by Trimmomatic version 0.38 [72] before alignment to the published Atlantic salmon reference genome http://ftp.ensembl.org/pub/release-99/gtf/salmo_salar/ using STAR version 2.7.0 [73]. In the gtf file, four tandemly duplicated medium-wavelength sensitive visual opsins were annotated as one gene (ENSSSAG00000055587) and two long-wavelength sensitive visual opsins were annotated as (ENSSSAG00000010443). To include all six genes the gtf file was edited. Further, Samtools version 1.6 [74] were used to process the output files from the aligner and counts were generated using HTSeq version 0.11.2 [75]. DESeq2 version 1.26.0 [76] was used to generate normalized counts and perform differential expression analyzes using the Wald statistical model. Two arrays of normalized counts were generated in DESeq2, namely (i) normalized counts for a specific developmental series (i.e., LDM 255 dd, 379 dd, 555 dd, 690 dd) and (ii) normalized counts for all samples at 690 dd (S1 and S2 Tables). For the sampling point 690 dd in the developmental series, the alevins sampled at 10:00 start and 10.00 end from the circadian study were chosen to get a n = 8. In the differential gene expression analyzes by DESeq2, genes with counts less than 10 per comparison were not included and the adjusted p-value was set to < 0.05. Pathway enrichment analyzes was performed by clusterProfiler version 1.13.0 [77], using the universal enrichment analyzer (enricher) with a pvalueCutoff = 0.05 and pAdjustMethod = "BH". Analyzes of the resulting list of GO terms were conducted in QuickGO [78,79] to reveal the association between GO terms. The GeneIDs listed for each GO terms were evaluated to reveal if associated GO terms shared GeneIDs. The condensed list of the 55 ontologies exclusive in LL vs LDM (Fig 5) were made by including the overarching ontology among the associated GO terms. Venn diagrams were created using Venny v2.1 [80] and plots were made by ggplot2 v3.3.2 [81]. The Kyoto Encyclopedia of Genes and Genomes (KEGG) phototransduction pathway figures were generated by mapping the KEGG annotated differentially expressed genes to KEGG phototransduction pathway as described in the KEGG Mapper tool [82].

### Detection of nonvisual opsin genes in the Atlantic salmon and heatmaps of nonvisual opsins and clock genes

*In silico* analyzes of the Atlantic salmon genome database [12] were performed by BLASTP or BLASTN on NCBI [83] using zebrafish nonvisual opsin sequences as a bait [9] to generate an list of the nonvisual opsins in Atlantic salmon for determine the photoreceptive capacity. In addition, the results were compared with information from [10] and [22]. In total, 42 intact nonvisual opsins were determined, with corresponding GeneIDs and chromosome locations listed in S3 Table. The table also notes the Ss4R pairs denoted as ohnologues, paralogues formed by a whole genome duplication event. These were detected by confirming that the paralogues were in the corresponding homologues region, which are subdivided into 98 collinear blocks along the 29 chromosomes of Atlantic salmon [12]. Note that *tmtopsin3a2* (LOC106568690) is not annotated and *opn9* (LOC106576962) is not annotated correctly within the Ensembl database [84], these were therefore not included in the heatmaps. Heatmaps of the normalized counts of nonvisual opsins and clock genes [38] during the different developmental stages were made by the pheatmap v1.0.12 [85].

### Identification of cycling gene expression profiles

Cycling transcripts were identified by JTK_CYCLE v3.1 in R, which is designed to efficiently identify and characterize cycling variables in large collection of data, such as genome-scale data sets. JTK_CYCLE v3.1 effectively distinguishes rhythmic and non-rhythmic transcripts by determining *P*-values based on Kendall’s rank correlation coefficient statistical analyzes [86]. Normalized counts form the circadian sampling dataset (LDM at 690 dd) were used as input data to the JTK_CYCLE script, where the “period” parameter was set to “5:7” to identify genes that oscillate every 20–28 h (sampling interval 4 h). The parameters were set to identify additional significantly cyclical genes due to the unknown level of clock entrainment and the nature of artificial environments.

## Supporting information

S1 Fig Experimental setup and spectral properties of the light-emitting diodes (LED) arrays. A) Schematic illustration of the experimental setup showing constant or changing light environments. Embryos and alevins were exposed to different lighting regimes from fertilization to first feeding (121 days). The experiments were divided across three parameters: (i) different photoperiods (i.e. continuous white light (LL), continuous darkness (DD), or a white light:dark (LD) cycle of 14:10); (ii) intensities of LD white light, consisting of high (LDH), medium (LDM) or low (LDL) light levels; (iii) a LD cycling condition with medium intensity of light of different wavelengths of the visible spectrum, namely (blue (LDB), green (LDG), red (LDR). The arrows indicate sampling points during development, 255 dd (40 days), 379 dd (60 days), 555 dd (90 days) and 690 dd (113 days). The circadian sampling for LDM at 690 dd is highlighted by wider boxes indicating the sampling points for the 24 h series. All light regimes were sampled at 18:00 and 02:00. B) Spectrum of the white LED, warm white 2700K (λ_max_ = ~610 nm), at medium intensity and the narrow bandwidth spectrum of blue (λ_max_ = ~450nm), green (λ_max_ = ~535nm), and red (λ_max_ = ~660nm) LEDs. C) Pictures of eggs in the egg incubators with medium white, blue, green, and red light.
(TIF)

S2 Fig Developmental series. A) Venn diagram of all genes expressed at different developmental stages, only genes that had a total count greater than 10 within a developmental stage were included. Only a few genes are unique for each color-coded developmental stage (255 dd, violet; 379 dd, yellow; 555 dd, green; 690 dd, red) and 33,377 genes (87.5%) being expressed at all four stages. B) Bar chart of differentially expressed genes (DEGs), comparing 255 dd, 379 dd and 555 dd to 690 dd. C) Venn diagram of Gene Ontology (GO) terms comparing 255 dd, 379 dd and 555 dd to 690 dd. The diagrams show that 51.3% of upregulated and 43% downregulated terms are shared between the different comparisons.
(TIF)

S3 Fig Heatmap of nonvisual opsin gene expression in Atlantic salmon. The heatmap is shown by individual normalized counts, scaled by row.
(TIF)

S4 Fig Heatmap of circadian clock gene expression in Atlantic salmon. The heatmap is shown by individual normalized counts, scaled by row.
(TIF)

S5 Fig A logfold2 change heatmap of nonvisual opsins and circadian clock genes in Atlantic salmon. The heatmap was made by comparing the mean count for each developmental stage.
(TIF)

S6 Fig Differentially expressed genes apparent in the Kyoto Encyclopedia of Genes and Genomes (KEGG) phototransduction pathway. Comparing A) continuous darkness (DD) B) periodicity of blue light (LDB) C) periodicity of green light (LDG) D) periodicity of red light (LDR) to periodicity of white light (LDM). The scale indicates the logfold2 change and the color code indicate upregulated (blue) and downregulated (red) genes.
(TIF)

S1 Table Normalized counts for the developmental series. The normalized counts are for the LDM at 255 dd, 379 dd, 555 dd and 690 dd.
(XLSX)

S2 Table Normalized counts for the samples at 690 dd. The table contains the normalized counts for the LDM circadian series and day and night samplings under DD, LL, LDH, LDL, LDB, LDG and LDR conditions.
(XLSX)

S3 Table The full component of nonvisual opsin genes found in the genome of Atlantic salmon. The LOC-ID, Ensembl GeneID, chromosome location, strand and Ss4R duplication pair are included. Note that *tmtopsin3a1* is not annotated and *opn9* is incorrectly annotated in the Ensembl database. These genes are identified by LOC-ID.
(XLSX)

S4 Table Developmental series, pathway enrichment analyzes. Number of differentially expressed genes (DEGs) and pathway enrichment analyzes comparing 255 dd, 379 dd, and 555 dd to 690 dd, listing both upregulated and downregulated Gene Ontology (GO) terms together with Ensembl GeneIDs for each DEGs.
(XLSX)

S5 Table Mean normalized counts of the nonvisual opsins and circadian clock genes for the developmental series. The counts were obtained from S1 Table, and the mean was calculated and used for the heatmaps in Fig 1.
(XLSX)

S6 Table Cycling genes in the circadian series. Results from JTK_CYCLE (p < 0.05) showing cycling genes. Lines indicate the (p < 0.01) and (p < 0.001) and PER show the periodicity of 20 h, 24 h or 28 h.
(XLSX)

S7 Table Number of differentially expressed genes (DEGs) by DESeq2. In the circadian series the different sampling points were compared to either 10:00, 02:00, 18:00 or an altering control and the number of DEGs are listed. The table also include the upregulated or downregulated DEGs from the analyzes within a light stimulation (02:00 vs 18:00) and comparing different light conditions to DD, LL or LDM at 690 dd (shown in Fig 3).
(XLSX)

S8 Table Pathway enrichment analyzes. The result of clusterProfiler for differing photoperiods (DD, LL compared to LDM) and wavelength experiments (LDB, LDG, LDR compared to LDM).
(XLSX)
