# Supplementary figures and images for: Photoreception and transcriptomic response to light during early development of a teleost with a life cycle tightly controlled by seasonal changes in photoperiod
Source: PLoS Genet. 2022 Dec 12;18(12):e1010529. doi: 10.1371/journal.pgen.1010529 (PMC9744326; doi:10.1371/journal.pgen.1010529)

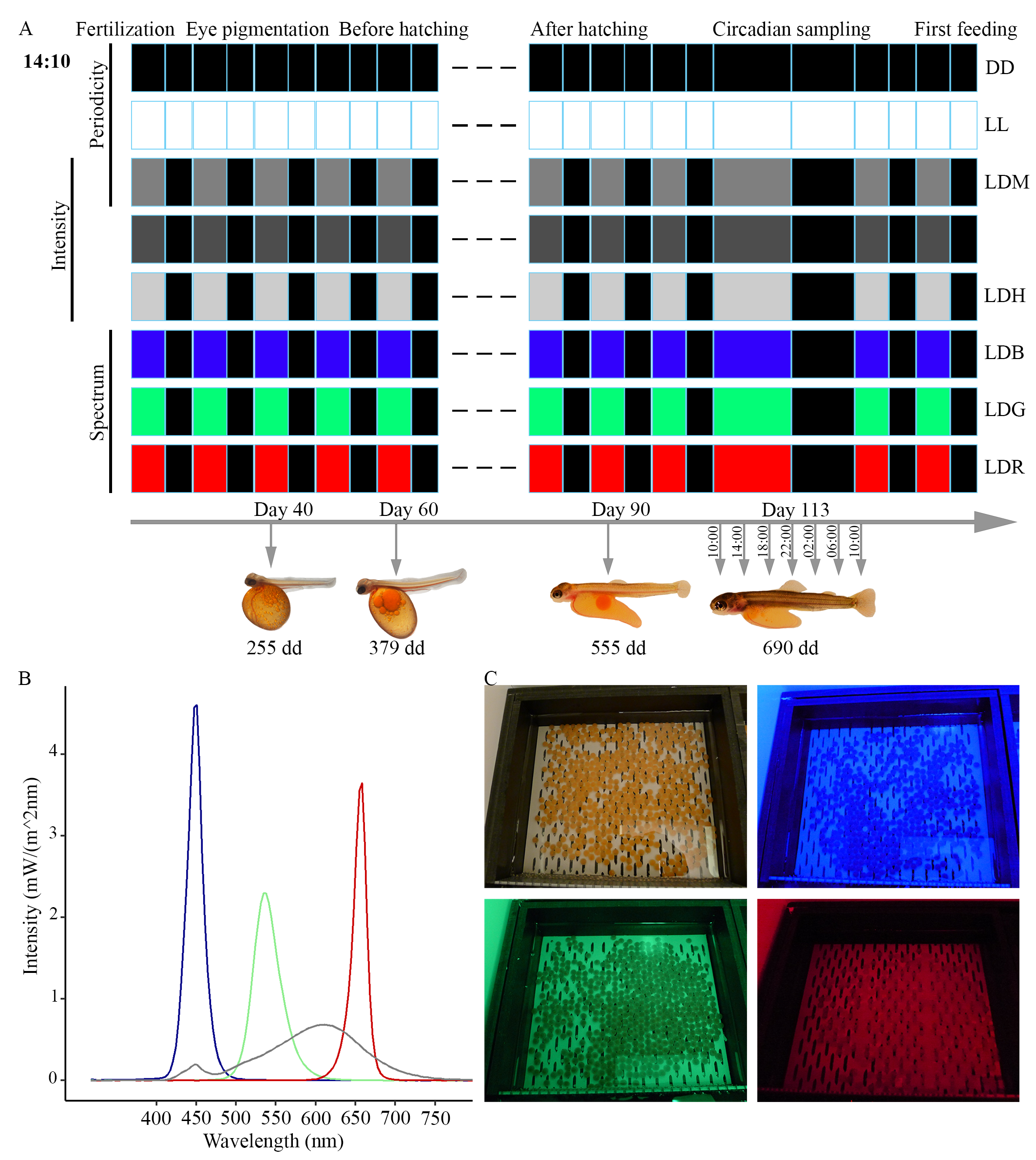

Supplement: S1 Fig — A) Schematic illustration of the experimental setup showing constant or changing light environments. Embryos and alevins were exposed to different lighting regimes from fertilization to first feeding (121 days). The experiments were divided across three parameters: (i) different photoperiods (i.e. continuous white light (LL), continuous darkness (DD), or a white light:dark (LD) cycle of 14:10); (ii) intensities of LD white light, consisting of high (LDH), medium (LDM) or low (LDL) light levels; (iii) a LD cycling condition with medium intensity of light of different wavelengths of the visible spectrum, namely (blue (LDB), green (LDG), red (LDR). The arrows indicate sampling points during development, 255 dd (40 days), 379 dd (60 days), 555 dd (90 days) and 690 dd (113 days). The circadian sampling for LDM at 690 dd is highlighted by wider boxes indicating the sampling points for the 24 h series. All light regimes were sampled at 18:00 and 02:00. B) Spectrum of the white LED, warm white 2700K (λmax = ~610 nm), at medium intensity and the narrow bandwidth spectrum of blue (λmax = ~450nm), green (λmax = ~535nm), and red (λmax = ~660nm) LEDs. C) Pictures of eggs in the egg incubators with medium white, blue, green, and red light. (TIF) [file pgen.1010529.s001.tif]

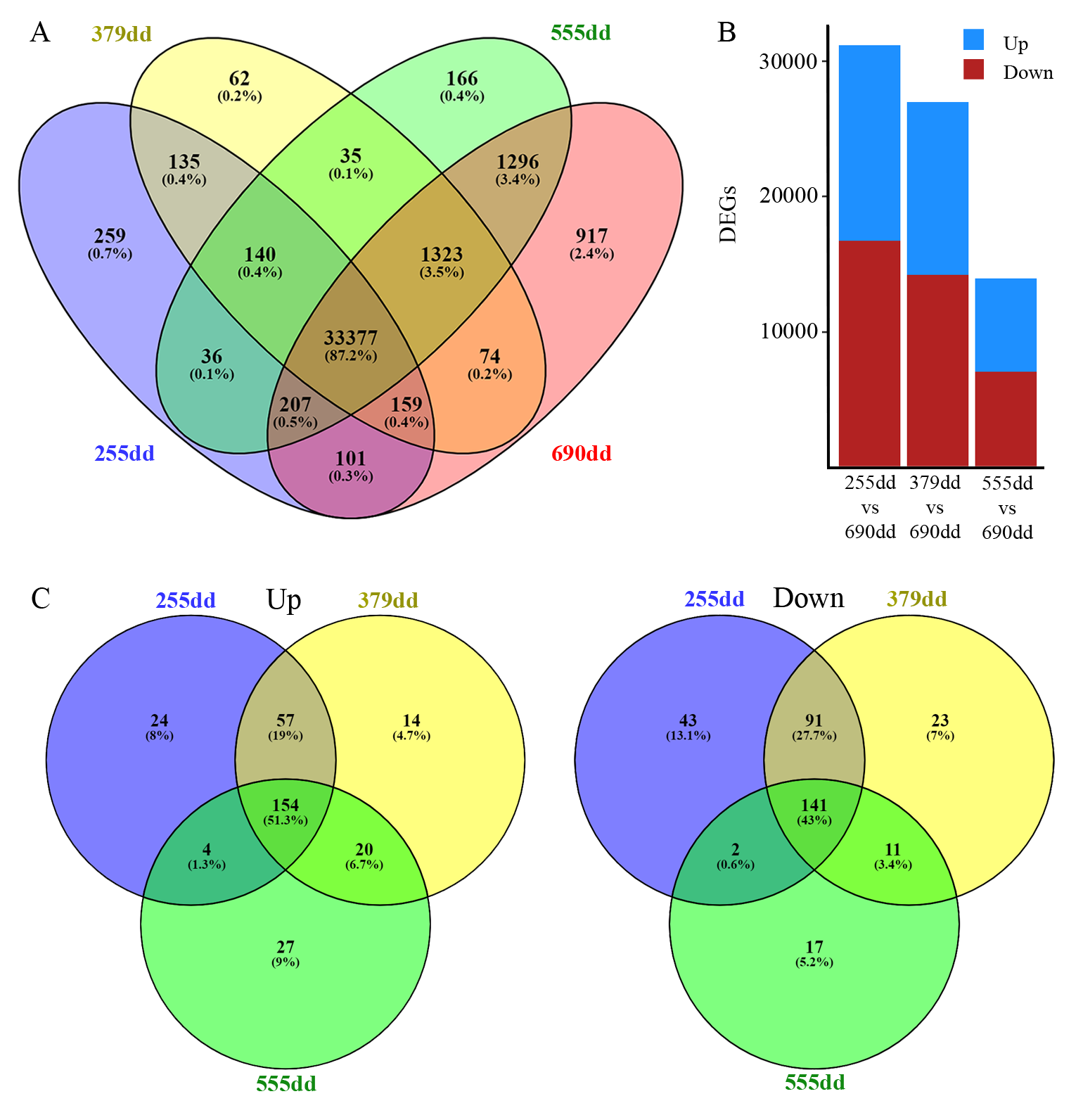

Supplement: S2 Fig — A) Venn diagram of all genes expressed at different developmental stages, only genes that had a total count greater than 10 within a developmental stage were included. Only a few genes are unique for each color-coded developmental stage (255 dd, violet; 379 dd, yellow; 555 dd, green; 690 dd, red) and 33,377 genes (87.5%) being expressed at all four stages. B) Bar chart of differentially expressed genes (DEGs), comparing 255 dd, 379 dd and 555 dd to 690 dd. C) Venn diagram of Gene Ontology (GO) terms comparing 255 dd, 379 dd and 555 dd to 690 dd. The diagrams show that 51.3% of upregulated and 43% downregulated terms are shared between the different comparisons. (TIF) [file pgen.1010529.s002.tif]

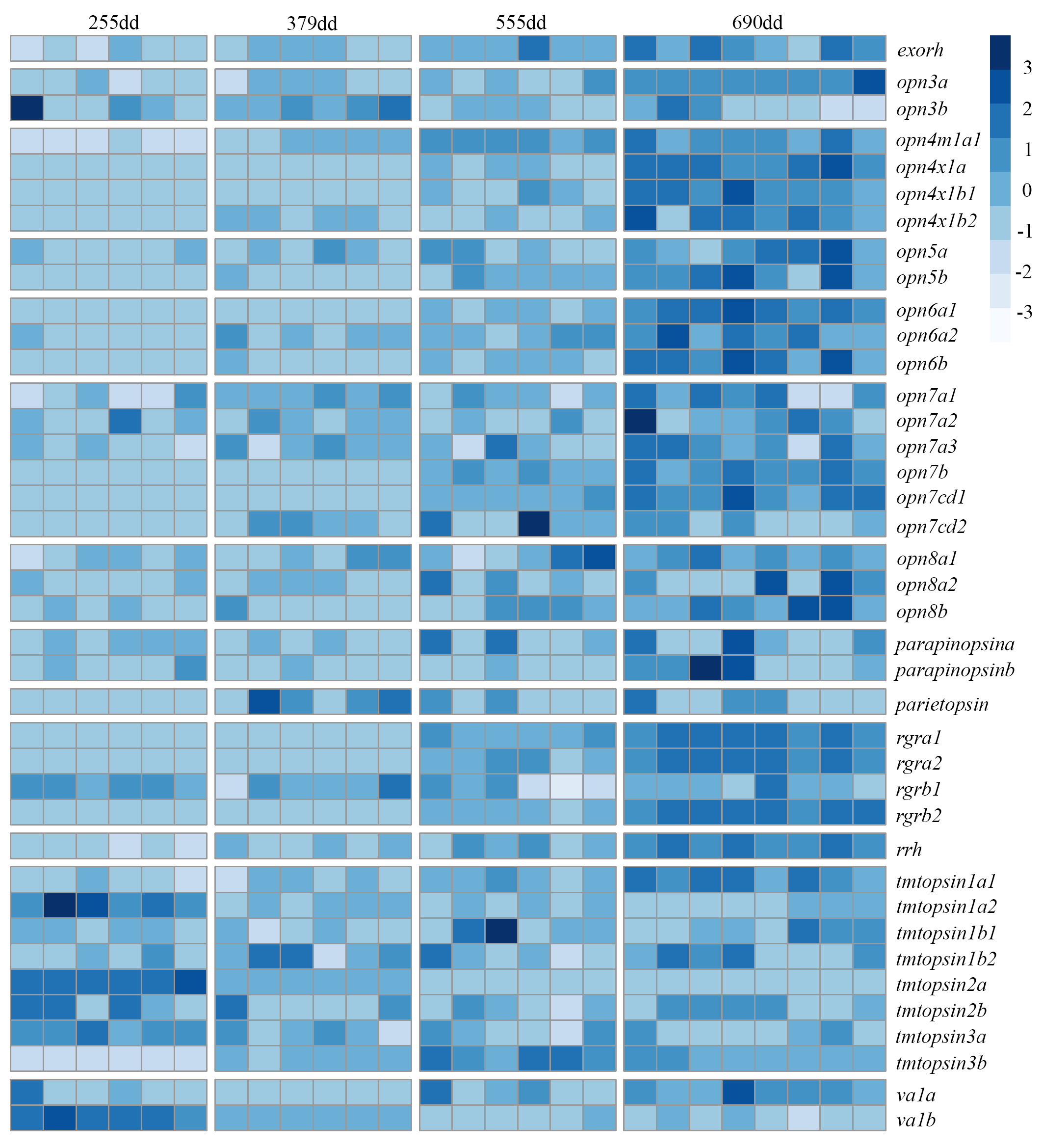

Supplement: S3 Fig — The heatmap is shown by individual normalized counts, scaled by row. (TIF) [file pgen.1010529.s003.tif]

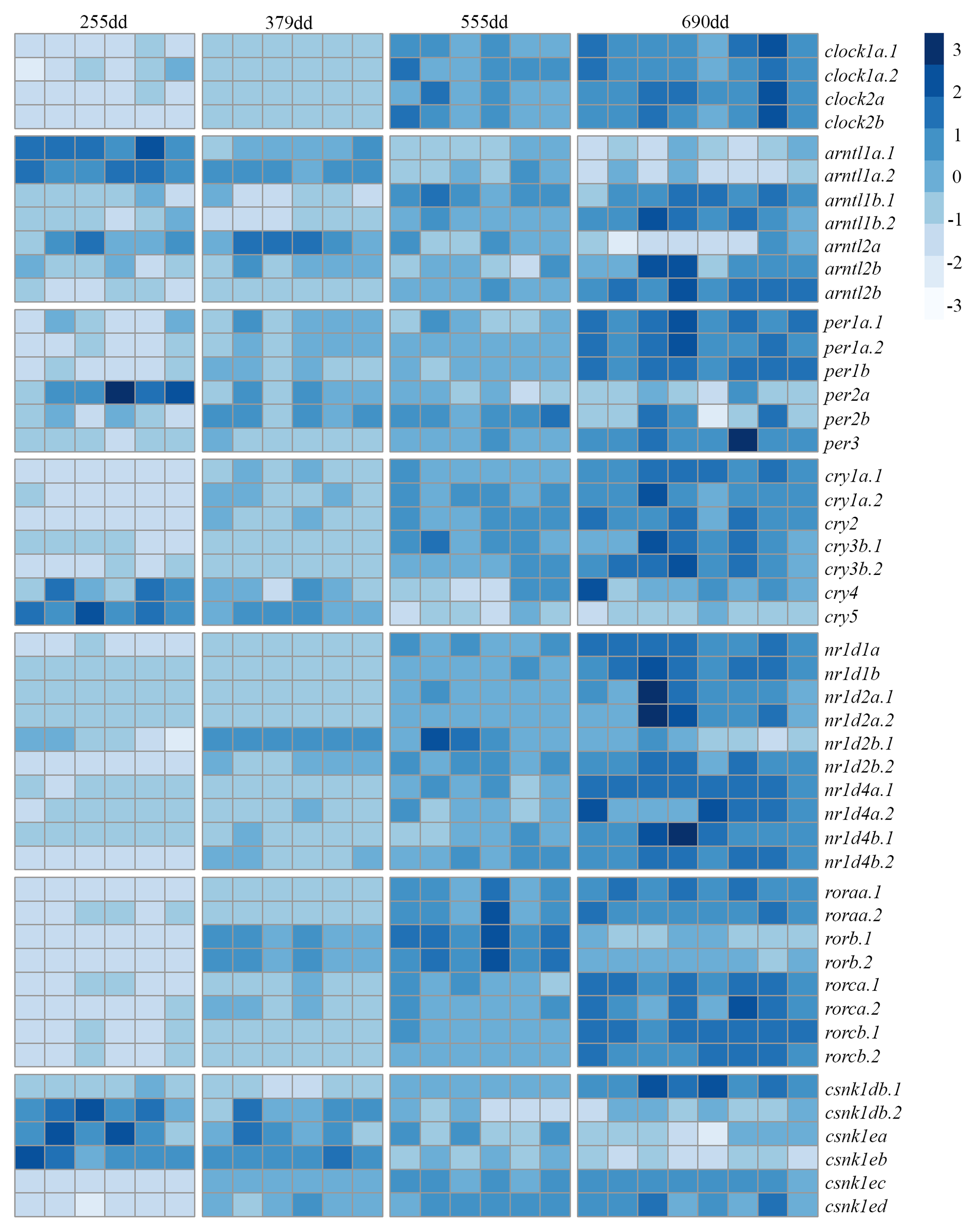

Supplement: S4 Fig — The heatmap is shown by individual normalized counts, scaled by row. (TIF) [file pgen.1010529.s004.tif]

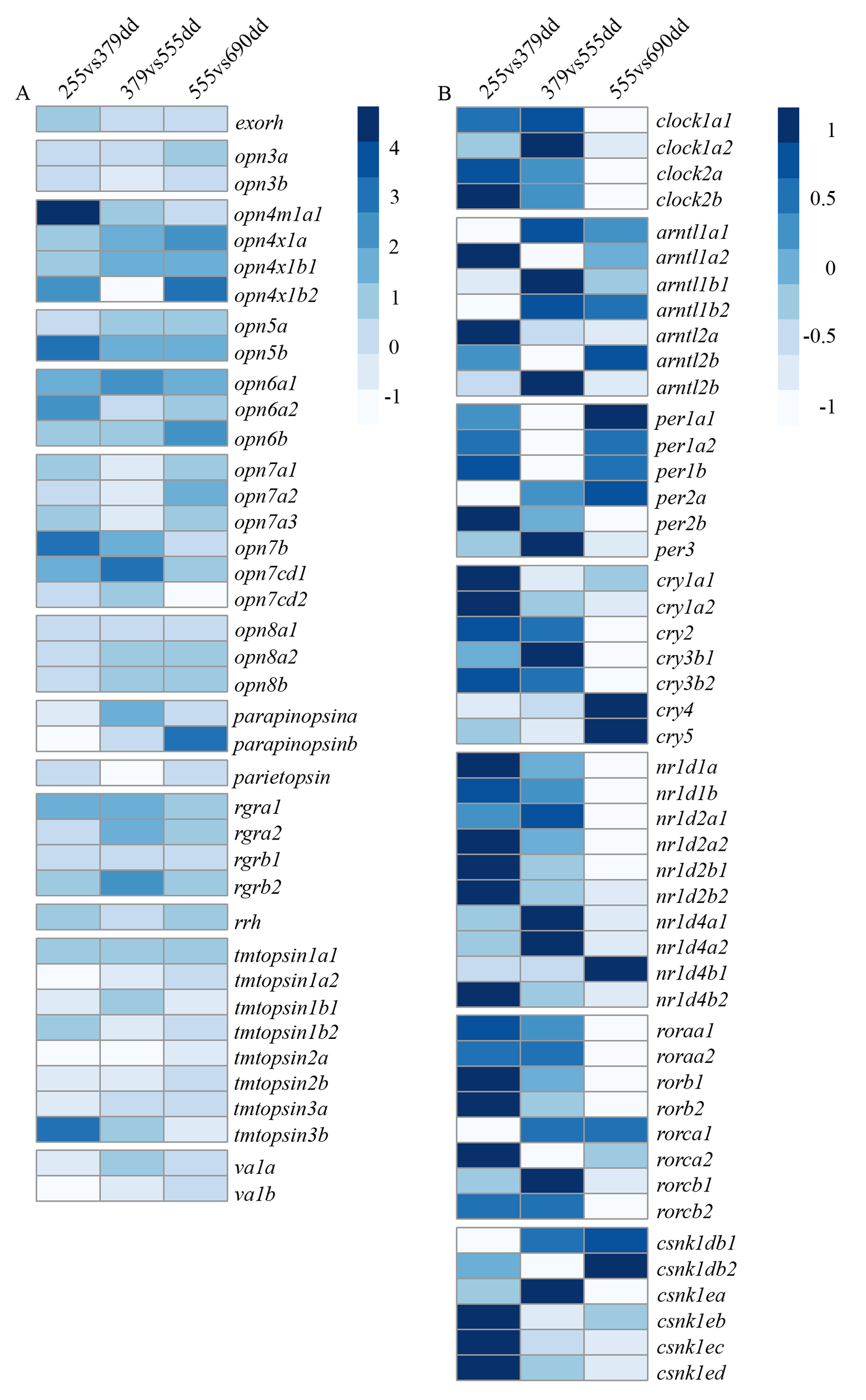

Supplement: S5 Fig — The heatmap was made by comparing the mean count for each developmental stage. (TIF) [file pgen.1010529.s005.tif]

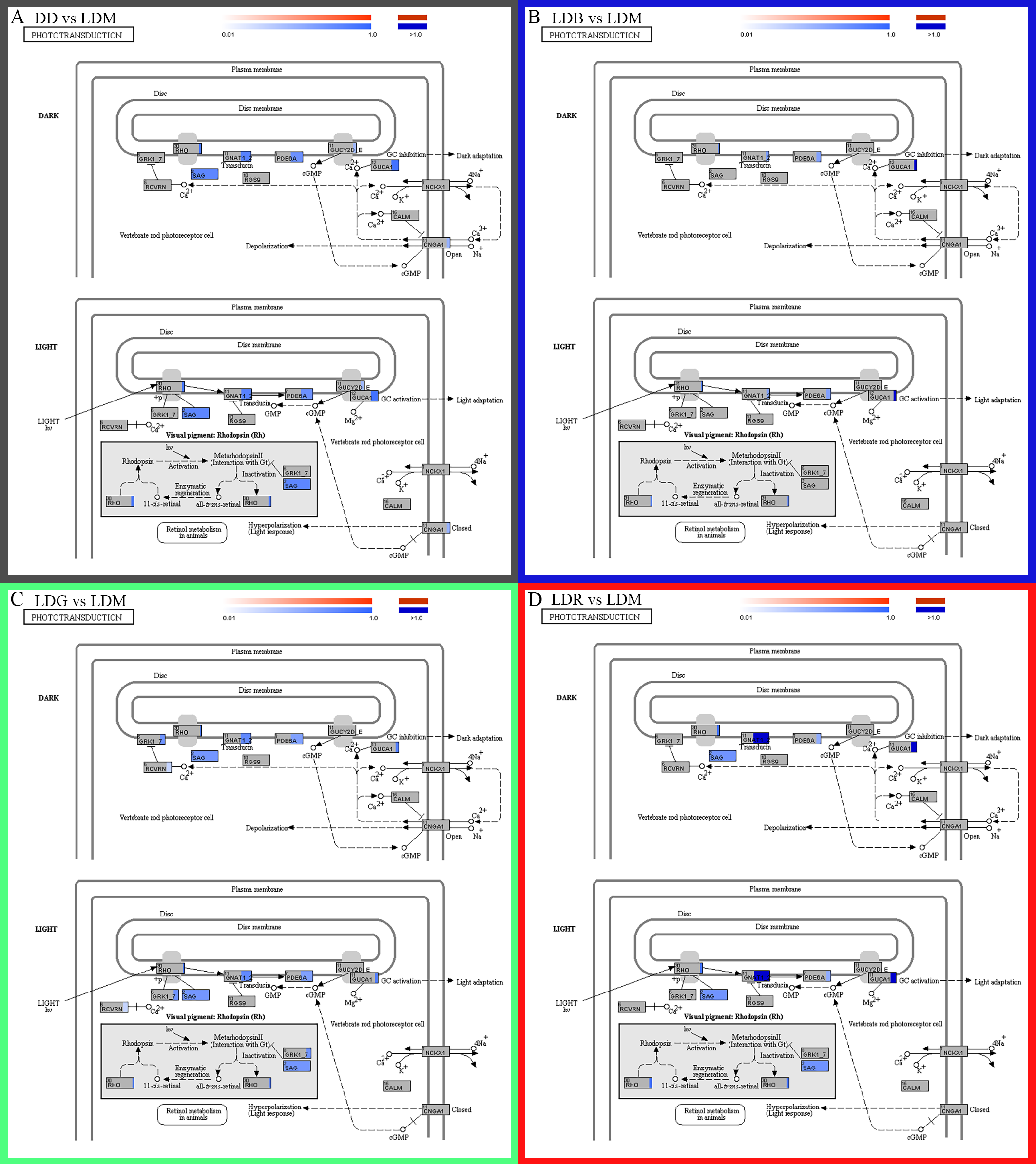

Supplement: S6 Fig — Comparing A) continuous darkness (DD) B) periodicity of blue light (LDB) C) periodicity of green light (LDG) D) periodicity of red light (LDR) to periodicity of white light (LDM). The scale indicates the logfold2 change and the color code indicate upregulated (blue) and downregulated (red) genes. (TIF) [file pgen.1010529.s006.tif]
